# Identifying Dietary Strategies to Improve Nutrient Adequacy among Ethiopian Infants and Young Children Using Linear Modelling

**DOI:** 10.3390/nu11061416

**Published:** 2019-06-24

**Authors:** Aregash Samuel, Saskia J. M. Osendarp, Elaine Ferguson, Karin Borgonjen, Brenda M. Alvarado, Lynnette M. Neufeld, Abdulaziz Adish, Amha Kebede, Inge D. Brouwer

**Affiliations:** 1 Ethiopian Public Health Institute (EPHI), Gulele Sub City, Addis Ababa, Ethiopia; amha.kebede@gmail.com; 2 Division of Human Nutrition and Health, Wageningen University and Research, 6700 AA Wageningen, The Netherlands; saskia.osendarp@wur.nl (S.J.M.O.); karin.borgonjen@wur.nl (K.B.); brenda.medina@gmail.com (B.M.A.); 3 Nutrition International (NI), Ottawa, ON K2P2K3, Canada; 4 London School of Hygiene and Tropical Medicine (LSHTM), London WC1E 7HT, UK; elaine.ferguson@lshtm.ac.uk; 5 Global Alliance for Improved Nutrition (GAIN), 1202 Geneva, Switzerland; lneufeld@gainhealth.com; 6 Nutrition International (NI), Nifas Silk Lafto Sub City, Kebele 04, Addis Ababa, Ethiopia; aadish@nutritionintl.org

**Keywords:** complementary food, Optifood analysis, nutrient adequacy, food-based dietary recommendations, Ethiopia

## Abstract

Nutrient adequacy of young children’s diet and best possible strategies to improve nutrient adequacy were assessed. Data from the Ethiopian National Food Consumption Survey were analysed using Optifood (software for linear programming) to identify nutrient gaps in diets for children (6–8, 9–11 and 12–23 months), and to formulate feasible Food-Based Dietary Recommendations (FBDRs) in four regions which differ in culture and food practices. Alternative interventions including a local complementary food, micronutrient powders (MNPs), Small quantity Lipid-based Nutrient Supplement (Sq-LNS) and combinations of these were modelled in combination with the formulated FBDRs to compare their relative contributions. Risk of inadequate and excess nutrient intakes was simulated using the Estimated Average Requirement cut-point method and the full probability approach. Optimized local diets did not provide adequate zinc in all regions and age groups, iron for infants <12 months of age in all regions, and calcium, niacin, thiamine, folate, vitamin B12 and B6 in some regions and age-groups. The set of regional FBDRs, considerably different for four regions, increased nutrient adequacy but some nutrients remained sub-optimal. Combination of regional FBDRs with daily MNP supplementation for 6–12 months of age and every other day for 12–23 months of age, closed the identified nutrient gaps without leading to a substantial increase in the risk of excess intakes.

## 1. Introduction

Ensuring optimal Infant and Young Child Feeding (IYCF) practices has been identified as one of the most effective public health interventions to improve child survival in developing countries [1]. The United Nations International Children’s Emergency Fund (UNICEF) and the World Health Organization (WHO) recommends that infants are exclusively breastfed during the first six months of life and are given nutrient-dense semi-solid or solid complementary foods in addition to continued breastfeeding from the age of six months until at least two years of age [2]. In its 2003 Global Strategy for IYCF, the WHO emphasizes the use of suitable locally available foods when introducing complementary foods [3]. This recommendation is challenging in a country like Ethiopia, where children transition directly to adult diets that are often monotonous, and primarily composed of low nutrient-dense cereal-based foods. Further, any infant-specific foods fed to young children tend to be of low nutrient density [4,5,6,7].

According to Ethiopia’s 2016 Demographic and Health Survey, more than half of children 6–23 months of age do not achieve the recommended feeding frequency for their age and just 7% of these children consume a minimum acceptable diet (a combination of minimum dietary diversity which is a consumption of four or more food groups from the seven food groups and minimum meal frequency which is consumption of two or more (at age 6–8 months), three or more (at age 9–23 months) solid or semi-solid feeds for breastfeeding children or four or more solid or semi-solid or milk feeds for non-breastfeeding children at age 6–23 months) [8,9]. Data from the 2011 Ethiopian National Food Consumption Survey (NFCS) reported high intakes of iron across all age groups including children from 1 to 3 years of age [10], whereas intakes of other micronutrients such as zinc and vitamin A were below recommendations. Although several small-scale and short term Infant and Young Child Nutrition programs combining capacity building and behaviour change communication were able to improve IYCF practices in Ethiopia [11,12], they are limited by the absence of evidence-based, realistic food-based dietary recommendations (FBDR) to guide improved practices. One of the few examples using realistic guidelines and behaviour change communication (BCC) to improve IYCF and growth is the cluster randomized trial in Ethiopia by Kang et al. (2017), demonstrating the effectiveness of including guideline-based BCC in existing government programmes in improving linear growth among children living in resource poor settings [13].

Linear programming is a mathematical method that has been used to formulate robust FBDR [14,15,16,17,18]. Linear programming has also been used to develop different FBDRs for different contexts, and to objectively identify key nutrient gaps in optimized local diets [17,19,20,21] and to define “problem nutrients” i.e., nutrients for which it may be difficult to ensure nutrient adequacy with local foods alone [18,22]. In Ethiopia, the Alive and Thrive programme used linear programming to determine whether micronutrient requirements of breastfed infants (6–8 and 9–11 months) could be met using only unfortified local foods, and illustrated the nutritional needs of infants were difficult to meet when fortified products are not consumed [23]. These analyses, however, were limited to a pilot study conducted in one region, using a list of foods available in markets rather than information on foods that were actually consumed by infants in this region. Additionally, they did not take into account the regional variability in food consumption patterns in Ethiopia.

To address these limitations, in the current study, we used nationally representative individual dietary intake data from the NFCS [10] in linear programming analyses, to identify “problem nutrients” and formulate realistic FBDRs for young children (6–8, 9–11, and 12–23 months of age) from four regions of Ethiopia. In addition, in combination with the formulated FBDRs we modelled various nutrition intervention alternatives that could be used to help improve nutrient adequacy: including a locally produced complementary food (CF),and Micronutrient Powders (MNPs) [24] and Small quantity Lipid-based Nutrient Supplements (Sq-LNS), both in combination with and without CF [25,26]. In addition, we also assessed the risk of inadequate iron and zinc intakes and excess intakes through these interventions. Analysis of inadequate and excess intake of iron and zinc was considered due to the concern of risk for too high iron intake as reported by NFCS [10]. Furthermore, we included zinc due to the high prevalence of stunting [8] and low observed dietary zinc intake in young children [27]. Ethical approval for the NFCS was obtained from the Scientific and Ethics Review Committee of Ethiopian Public Health Institute (EPHI), reference number EHNRI 6.13/157.

## 2. Methods

### 2.1. Study Population

In this secondary data analyses, we used anthropometric and 24 h dietary recall data collected from a subgroup of 6–23 months old children in the cross-sectional NFCS [10]. The NFCS data were collected between June–September 2011. The NFCS is a nationally and regionally representative sample of 6–35-month old children (n = 8079). Our analyses were performed on a subgroup of 31% of these children (n = 2498) by only including children within the age range of 6–23 months and residing in four regions Tigray, Amhara, Oromia and South Nations, Nationalities and Peoples Region (SNNPR). These four regions were selected to ensure representation of regions included in the pilot complementary feeding program of Nutrition International (NI) and UNICEF [28]. Moreover, these regions are the largest regions in the country and represent different local cultures and child feeding habits and practices.

### 2.2. Optifood Analysis

We used linear programming (LP) software (Optifood) to develop Food-Based Dietary Recommendations (FBDR), identify nutrient gaps in local diets and test alternative interventions, as reported elsewhere [16,29,30]. The LP analyses were done by age group and region to theoretically determine whether (1) FBDR could ensure nutrient adequacy (FBDR) and if not, whether including in the set of FDBRs (2) a locally produced CF consisting of grains and legumes (FBDR + CF), (3) MNP (FBDR + MNP), (4) Sq-LNS (FBDR + Sq-LNS), (5) CF and MNP (FBDR + CF + MNP) or (6) CF and Sq-LNS (FBDR + CF + Sq-LNS) would further improve the nutrient adequacy of young children’s diets.

#### 2.2.1. Data Preparation

Data were prepared by region and age group (6–8 months, 9–11 months and 12–23 months). We defined model parameters based on the information on food intakes and recipe composition of NFCS data, using the Optifood data preparation programme in MS Access. Constraints used to ensure realistic modelled diets were defined by (i) the average energy requirement for the target groups, estimated using the FAO/WHO algorithm for energy requirement and the standard average weight for children in each age group as reported by WHO Child Growth Standards: 7.9 kg for 6–8 months, 8.8 kg for 9–11 months and 10.5 kg for 12–23 months children [31,32]; (ii) foods commonly consumed by the target population defined as those consumed by more than 3% of the target population (per region and age group), excluding water, condiments and salt. Foods with a portion size below 0.5 g/day or consumed with a weekly frequency below 0.5 servings per week were also excluded. Although most studies using Optifood analysis use a cut-off point of more than 5%, it was necessary in our study to use 3% to meet the energy requirements sufficient for Optifood software to model diets; (iii) the estimated average serving size of those foods, calculated as the daily median intake of foods in grams for only children consuming that particular food, and (iv) the minimum and maximum consumption frequency per week for each food, assigned food group and sub-food group. Foods were assigned to food groups and subgroups (see Supplemental Table S1). For each (sub) food group these were defined as the 10th and 90th percentiles of weekly frequencies, respectively. For each food, the maximum number of servings per week was based on the proportion of children consuming each food, while the minimum number of servings/week per food were usually zero except for breast milk intake. The median weekly consumption frequency for food groups defined the food pattern goals used in subsequent steps in Optifood; (v) staple foods were identified as foods belonging to the food groups’ grain and grain products or starchy roots. Snacks were defined as foods consumed only in between meals. The type of meal (snack or staple) was determined based on the nature of the food and time of the food consumption and (vi) the minimum and the maximum number of daily servings per week of breastmilk were set at 6.9 and 7.1 respectively. The portion sizes and the nutrient composition of breast milk used are explained in Section 2.3.

The list of nutrients considered in Optifood models were protein, fat, calcium, vitamin C, vitamin B_1_, vitamin B_2,_ vitamin B_6,_ niacin, vitamin A, folate, vitamin B_12,_ iron and zinc. The content of vitamin A, iron, zinc, calcium, protein, fat, carbohydrate of foods consumed were obtained from the food databases compiled for NFCS 2011, which were primarily from local food composition table (FCT) III and IV [33,34] and other regional or international published data [10]. Food composition values for vitamin B_6_, B_12_ and folate were derived from the United States Department of Agriculture (USDA) food composition database. The FAO/WHO daily nutrient requirements for protein, thiamine, riboflavin, niacin, vitamin B_6_, folate, vitamin B_12_, vitamin C, vitamin A, calcium, iron, and zinc were used [35,36,37]. As the diet is mainly cereal-based with low consumption of animal-derived products and vitamin C, and the dietary diversity of children is extreme low, [38,39] we considered low bioavailability (15%) for zinc [10] and 5% bioavailability for iron [10].

#### 2.2.2. Analysis Using Linear Programming

The linear programming analyses were done by age group (n = 3) and region (n = 4) using Optifood [15,16], to develop 12 sets of FBDR. The analyses, for each group, were done in Modules II and III in Optifood. The model parameters, for these analyses, were set-up in Module I. Specifically, after the model parameters were entered (i.e., food lists, model constraints and goals), 16 linear programming models were run, in Module I (module to set-up model parameters). Each modelled diet had a different objective function. An expert who was familiar with local dietary patterns then examined the foods selected in these 16 optimized 7-day diets to decide whether at least some individuals from the population would consume them. Model parameters were then modified until they resulted in modelled diets that could be consumed by at least some people in the population. At this point, model parameters were locked and the analyses began. The first analyses were done in Module II (module to identify food-based recommendations) which selected the two 7-day optimized diets. Because these diets are the nutritionally best diets that can be selected given model parameters, they are used to guide selection of food-based recommendations (FBRs) to test in Module III. The objective functions for these two diets either minimized deviations below the RNI or minimized deviations below the RNI and away from the median food group patterns of the population. We examined the diet patterns of the first named optimized diet to select FBRs to test in Module III (module to test food-based recommendations), which included foods or food sub-groups that contributed ≥5% of at least 13 nutrients in the optimized diet and food groups that were higher in the optimized diet than the observed diet (median number of servings). In module III, two 7-day diets per nutrient were modelled (i.e., total of 26 diets) of which 13 minimized (worst-case scenario) and 13 maximized (best-case scenario) the nutrient content of the diet, for one nutrient, by preferentially selecting respectively the lowest and highest nutrient dense foods for that specific nutrient [16]. Module III was first run without testing any FBRs. If a maximized (best-case scenario) modelled diet did not reach 100% of its nutrient’s RNI, the nutrient maximized was considered a “problem” nutrient i.e., it was impossible to select a diet that achieved the RNI for this nutrient. The FBRs subsequently were tested individually in Module III by including a constraint on the minimum number of servings per week for that FBR (i.e., a food, food sub-group or food group) and then examining the minimized (“worst-case” scenario) Module III diets for each nutrient to determine the percentage RNI achieved in the diet. These “worst-case” scenario levels simulate the lowest values in the nutrient’s intake distribution; which if ≥EAR predict a low percentage of the population would be at risk of inadequate intakes. If an individual FBR did not achieve ≥70% RNI for all nutrients, then combinations of individual FBRs were systematically tested (minimized diets in Module III). Finally, these combinations were examined and the set of FBRs with the highest number of nutrients reaching at least 70% of its RNI in the Module III “worst-case” scenario diets was selected as the baseline FBDR.

#### 2.2.3. Analysis of Alternative Options Using Linear Programming

When the selected baseline FBDR did not ensure a nutrient content of ≥70% in the worst-case scenario, 5 alternative options to FBDR were assessed to see whether nutrient gaps could be filled. In combination with the FBDRs, these 5 alternative options were a local CF product, MNP, Sq-LNS, CF + MNP and CF + Sq-LNS. The frequency per week of each option was also modified and tested. The best option was selected, for each age group and region, based on its %RNI in the Module III “worst-case” scenario diets. To define the nutrient composition of the local CF, which was one of the alternative interventions tested, we used a combination of the most abundant cereals and legumes available in the four study regions of Ethiopia according to an assessment of community-based production of complementary foods in Ethiopia [11]. We estimated the portion size of an average CF serving by identifying portion sizes as estimated by Lutter and Dewey (40 g for 6–11 months and 60 g for 12–23 months) [40] and, verifying these portion sizes for the different age groups with a group of mothers of children aged 12 to 23 months of age participating in an on-going MNP effectiveness study [41]. The nutritional composition used for the local CF per 100 g is shown in Table 1.

The Micronutrient Powder (MNP) used in our analyses was the Mix Me^®^ Vitamin and Mineral Powder from DSM Nutritional Products [42]. It contains a mixture of 15 vitamins and minerals in a single dose 1 g sachet (Table 1). We chose for a low-iron dose MNP (6 mg iron instead of recommended 12.4 mg iron) based on the concern in Ethiopia regarding necessity and safety of additional iron interventions due to the high iron intakes found in the Ethiopian National Food Consumption Survey of 2013 [41].

The Sq-LNS composition used in this study corresponded to the Nutributter^®^ composition from Nutriset. This supplement is formulated for children aged from 6 to 24 months old. The recommended dosage is 20 g/day to provide daily needs of 22 vitamins and minerals plus protein and essential fatty acids (Table 1). We chose this alternative intervention as the prevalence of stunting in Ethiopia is high and energy-dense supplementation may be needed [8,43].

### 2.3. Analysis of Inadequate and Excess Intakes

As the NFCS did not assess the quantity of breastmilk intake, we assumed an average daily intake of breast milk as reported by WHO for developing countries (660 g, 616 g and 549 g per day for 6–8 months, 9–11 months and 12–23 months children respectively) [22]. The nutrient composition of breast milk used was derived from WHO [44]. “Compl-eat© (version 1.0, Wageningen University, The Netherlands)” was used to calculate observed intakes of iron and zinc from CF and breast milk combined. Log transformation and square root transformation were used for intakes since nutrient intakes were not normally distributed. Adjusted observed intakes were then determined with the transformed data, using the Ugandan estimates for the within-person variation, since these estimates were not available for Ethiopia [45] and the between-person variation calculated from the NFCS, using the National Research Council (NRC) method [46,47], as described elsewhere [48].

The prevalence of inadequate and excess intakes of iron and zinc were calculated in 3 series of analyses; (1) the adjusted observed dietary intakes, (2) the adjusted observed intakes plus a daily (7 servings/week) or (3) every other day (3.5 servings/week) dosage of MNP. We used the Estimated Average Requirement (EAR) cut-point method for zinc (15% and 30% bioavailability) and the full probability approach for iron using a bioavailability of 5% and 10% for each age group [48,49], due to the skewedness of distribution of iron intakes. The EAR and the tolerable upper intake level (UL) from the Institute of Medicine (IOM) [50] were used for iron except for the EAR of 12–23 m which is from WHO/FAO [51]. For zinc, we used the EAR set by IOM [50] for 6–11 m and WHO/FAO [51] for 12–23 m and, used the UL suggested by WHO [52] as well as the UL suggested by IZiNCG [52], since the two UL cut-offs are quite different.

### 2.4. Other Analyses

The NFCS anthropometric data were analysed using WHO Anthro software version 3.2.2 [53] to estimate Z-scores for height-for-age (HAZ), weight-for-height (WHZ) and weight-for-age (WAZ). Children were classified as stunted, wasted and underweight if their Z-score values for HAZ, WHZ and WAZ were below-2 SD, respectively.

## 3. Results

The socio-demographic characteristics and nutritional status of the study children are presented in Table 2. Most children were 12–23 months of age and from rural areas (81–90%). A higher percentage of stunting and underweight were observed in Tigray (43% and 31%) and Amhara (41% and 28%) compared to SNNPR (35% and 21%) and Oromia (34% and 26%) respectively. Wasting was highest in Oromia (14%).

### 3.1. Overview of Foods Consumed

The majority of children in all age groups (≥85%) were consuming breastmilk (Supplemental Table S2). In children 6–8 months of age, on average only 28 (out of 80) foods were consumed by >3% of the population. This increased to 38 (out of 93) food items for 9–11 months old children and 52 (out of 172) food items for 12–23 months old children. The total number of foods consumed by the study children and number of foods consumed by >3% of children per age group and region is summarized in Supplemental Table S2.

The list of foods consumed by >3% of the children including the median serving sizes modelled, per age group and region is summarized in Supplemental Table S3. Among the grains, tef, wheat, sorghum and barley were consumed across all age groups and regions. The most commonly consumed legumes were peas, vetch, chickpeas, broad beans, and kidney beans. Milk was commonly consumed in all regions. It was observed that infants were rarely fed fruits or sweetened snacks, vegetables and eggs.

Median serving sizes ranged from 1–307 g/day (oil and buttermilk, respectively) for infants 6–8 months, 1–267 g/day for infants 9–11 months (oil and milk, respectively) and 1–234 g/day for children 12–23 months (oil and milk, respectively) and the actual types and amounts of foods consumed varied by region. For example, milk servings in Tigray were much smaller than those of other regions; biscuits or sweet cookies were only consumed by >3% of children in the Oromia region. Although similar grains or legumes were consumed across all regions, the serving sizes varied by region, see Supplemental Table S3. Fortified infant cereals were only included in the models in Tigray and Amhara regions because these food items were not consumed in the other regions. Eggs and starchy roots were not consumed in Amhara and Tigray regions respectively, while a starchy root like *enset* was only consumed in SNNPR and some parts of Oromia.

### 3.2. Problem Nutrients

Zinc was a common problem nutrient in all regions and across all age groups. Iron was a problem nutrient for infants from 6–11 months of age in all regions but not for the oldest (12–23 months) age group (see Table 3). Calcium was a problem nutrient for the youngest age group in all regions, except SNNPR, for the 9–11 month age group in Tigray and Amhara regions and for the 12–23 month age group only in Amhara region. Niacin was a problem nutrient across all age groups in all regions except for the youngest age group (6–8 months of age) in Tigray and the oldest age group (12 to 23 months) in SNNPR. Thiamine, folate, vitamin A, vitamins B_12_ and B_6_ were problem nutrients in some regions and age-groups, but not in all. The number of problem nutrients identified for children 12 to 23 months was greater than that of the younger age groups in Tigray and Amhara regions (see Supplemental Tables S4–S15).

### 3.3. Food-Based Dietary Recommendations

A set of 24 alternative food-based recommendations, reflecting commonly consumed foods, were selected and tested in Module III (worst-case scenario analyses). A summary of the FBDRs selected for each region per age group is presented in Table 4. These FBDRs do not include fruits because these foods were rarely consumed by the children and were not modelled (see Supplemental Table S3).

### 3.4. FBDR Combined with Local Complementary Food Products and Supplementation

Supplemental Tables S4–S15 show the worst-case scenario (Module III) of FBDR in combination with CF, MNP, Sq-LNS, or CF and MNP for different age groups and different regions. The addition of Sq-LNS to the FBDR was limited because of violation of the energy constraints. For example, in SNNPR for 9–11 months old children (Supplemental Table S14), we were able to add Sq-LNS to the FBDR 3.5 times per week (i.e., every other day) but its addition at a frequency of 7 times per week exceeded the energy constraints. Energy constraints also limited the addition of CF with Sq-LNS to the developed FBDR to just 2 servings/week. Similar results were found for the other regions and age groups (see Supplemental Table S4–S15).

**Figure 1:**
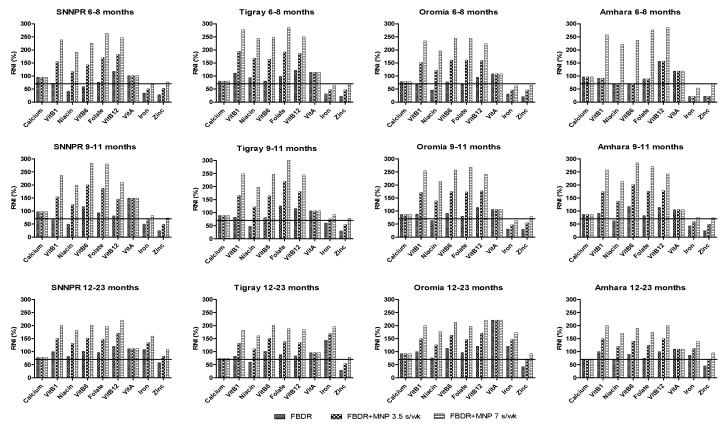
Minimized nutrient values (Module III) of different diet scenarios compared with Recommended Nutrient Intake (RNI) for 6–23 months in regions of Tigray, Amhara, Oromia and South Nations, Nationalities and Peoples Region (SNNPR).

There were regional differences in the ability of FBDR and MNP dosing regimens to ensure nutrient adequacy. For instance, FBDRs will likely ensure population-level nutrient adequacy for all nutrients except for zinc (all children), iron (6–11 months in all regions) and niacin (all 9–11 months, 6–8 months in Oromia and Tigray; and 12–23 months in Amhara). For children 9–11 months in Tigray, Amhara and SNNPR; and 12–23 months in Amhara and Oromia; 1 serving of MNP per day would be required to reach nutrient adequacy whereas 1 serving per 2 days would be sufficient for 12–23 months in Tigray and SNNPR. However, the four groups that would not reach nutrient adequacy for all nutrients even when MNP was included on a daily basis are 6–8 months in Tigray and Amhara; 6–8 months and 9–11 months in Oromia (Figure 1).

The prevalence of inadequate and excess intakes for iron and zinc are shown for the three age groups in Table 5. The prevalence of inadequate iron intakes at 10% bioavailability (between brackets at 5% bioavailability) was above 65% (80%) for 6–11 and above 40% (50%) for 12–23 months children, which was reduced to below 40% (76%) and 10% (36%) respectively, with simulated daily MNP provision. Similarly, for zinc at moderate bioavailability (between brackets at low bioavailability), the prevalence of inadequate intakes were above 90% (98%) for 6–11 and above 68% (96%) for 12–23 months old children, which were all reduced to 0% when simulated with a daily MNP provision, except for 12–23 months old children (53.9%) when using low zinc bioavailability. The prevalence of excess intakes was low, <6.5%, for all nutrients for infants <12 months, when the adjusted observed diet with or without provision of daily or every other day MNP were modelled using the WHO cut-off for UL. Prevalence of excess iron intake in children >12 months of age was <20% when only the adjusted observed diet was modelled. However, when simulating the provision of MNP every other day or daily, the prevalence of excess intakes of iron was above 20% in 12–23 months old children, whereas the prevalence of excess intakes of zinc was also above 20% with daily, but not every other day MNP supplementation, when the IZiNCG cut-off for UL was used. (See Table 5).

## 4. Discussion

Based on our assumptions and constraints set in Optifood analyses, the results of this study showed that for Ethiopian children 6–23 months of age, dietary improvements are possible using foods currently being consumed. However, even if FBDRs are fully implemented, our results indicated that nutrient requirements still will not be met, for all children, for some nutrients (“problem nutrients”), in particular for zinc in all age groups, iron in 6–8 months old children, and niacin in 9–11 months old children. These results suggest that to ensure nutrient adequacy for all children in these populations the developed local FBDR should be combined with the provision of special fortified complementary foods or nutrient supplements.

Daily MNP supplementation, in addition to the FBDR, made it possible to meet nutrient needs for nearly all nutrients, however, calcium requirements were not met because the MNP contains no calcium. For children from 12 to 23 months, decreasing the frequency of MNP consumption to one sachet every two days, in addition to FBDR, yielded a satisfactory nutrient content in all the three regions except Amhara where the zinc content of modelled diets remained low (53.9% of the RNI).

Adding Sq-LNS or a locally produced complementary food did not improve nutrient adequacy of the diet compared with FBDR alone. This likely occurred because these nutrient-dense foods replaced other nutritious energy-dense foods in the modelled diets, to avoid exceeding 100% of energy requirements in the model. We assume that in real life Sq-LNS interventions may still deliver substantial benefits to this population because (1) reported intake data from the NFCS suggested lower than recommended energy intakes for these age groups [10] and (2) we did not include cost constraints in the model and the best-modelled diets included some expensive food items. Sq-LNS may be a cheaper alternative for delivering additional energy and nutrients than the replaced food items. For instance, in two out of the four regions, fortified commercial infant cereals were reported to be consumed and were included in the model. When these fortified commercial infant cereals were not included in the model, the number of problem nutrients increased and only vitamin C, B_2_ and vitamin A met the criteria for nutrient adequacy in Module III (testing FBDR) (data are not shown). These findings highlight the importance and confirm the need for cost-effective measures, such as fortification or home-fortification, to improve the nutrient adequacy, especially for the youngest age group. Future research should investigate whether food fortification is a cost-effective strategy to increase dietary zinc intakes, and reduce the prevalence of zinc deficiency in this population [54].

We found that adding 3.5 or 7 servings per week of MNPs to the usual diets led to a decrease in the percentage of inadequate intakes for iron and zinc, without leading to a substantially increased risk of excessive intakes for iron. For zinc, daily MNP supplementation increased the risk of excess intakes to 51.0% of the population, which was reduced to <7% when the frequency of MNP use was reduced to every other day. These findings are in line with other studies confirming that in theory, the requirements of most, but not all, nutrients can be met by optimizing intakes of local foods [18,55]. Interventions, such as MNPs can be used to further improve nutrient adequacy [56,57]. However, concerns have been raised about possible side-effects of these interventions, including a possible, iron-induced increased morbidity from diarrhoea and other infectious diseases [58,59]. In addition, in a recent study simulating the effects of home-fortification of complementary foods in West Gojjam, Ethiopia, they observed a substantial increase in the risk of excessive intakes for iron and zinc in children 12–23 months of age [60]. These results are in contrast to our observations probably due to inter-study differences reflected in differences in the study population. There is uncertainty about tolerable upper intakes levels (UL), especially when bioavailability of iron and zinc are low in local diets [52,61]. Studies showed that bioavailability of iron and zinc depend on the intestinal health of the children, not only on the bioavailability from foods [62]. Factors resulting in impaired absorption of zinc also need to be considered, while addressing the risk of excess intake. Therefore, some caution is warranted when interpreting these findings [48]. Even so, our findings suggest that, for the older age groups (12–23 months of age), using the more conservative IZiNCG upper limits, distribution of MNP on every other day may be a safer choice.

The results of our analysis confirm that dietary habits differ across the different geographical regions in Ethiopia due to differences in cultural practices between regions [63,64]. For instance, eggs were not in the list of foods consumed by children from the Amhara region, most likely because animal source foods are not consumed during the long fasting season. In the other three regions, eggs could be recommended, at least for children 12–23 months of age. It is therefore advisable to develop separate food-based dietary recommendations for the different regions, taking into account differences across regions in food availability and consumption patterns. Regional differences in food intakes are known to exist in many countries.

In spite of the fact that the LP approach provides feasible and evidence-based results, this study has some limitations. First, in addition to the issue of the energy constraints described before, we calculated frequencies based on the percentage of the population who consumed each food because we did not have data on frequencies of food consumption. As the actual consumption frequency per food is more accurate than an estimated consumption using only one or two 24 h recalls, using estimated consumption frequency may affect model input data and lead to bias in nutrient adequacy of some nutrients. The extent of this bias is, however, not known. Second, the feasibility of implementing regional FBDR should be assessed by household trials in order to identify barriers and supporting factors for the adoption of FBDRs. A translation of these theoretical FBDR into practical guidelines should take into account the feasibility of these guidelines, by field-testing the FBDR in practice. In addition, increasing access to nutritious foods that are part of a healthy diet, but currently not consumed frequently enough to feature in the models, continues to be needed. Examples are fruits and vegetables, currently not included in the recommendations for the youngest children because they are not consumed frequently. Third, Optifood does not take into account all factors that affect food choices, such as variation in behaviour, food habits and the influence of social pressure on food choice. To some extent, the program takes this into account by using foods that are being consumed by at least 3% of the population. However, still, some of these foods may not be feasible options for part of the population, for example, fortified infant cereals which may be too costly. In general, it is known that the costs of diets based on nutritious local foods could be three to eight times higher than diets fortified with micronutrient powders (MNPs) [23] and costs were not included as a constraint in our analyses. Fourth, the intra-individual variation of the population was not quantifiable from survey data, to calculate the inadequate and excess intakes hence we used the variance from Uganda survey. However, the large number of survey days included provides a precise estimate of average intake at the population level which is advantageous for estimating median serving sizes [10,48,51]. Finally, the linear programming uses only the reported foods to develop the FBDR [65]. This restricts the use of foods that should be included in a healthy diet, such as vegetables and seasonal fruits. The FBDRs developed in this paper were based on food intake data of one season, during the period considered to be the longest lean season in Ethiopia characterised with low availability of a variety of foods [10]. This has affected the foods included in the Optifood modelling and, hence, the foods included in the FBDRs, especially vegetables and fruits. On the other hand, we consider using only consumed food a strength of the study because the use of consumed food would facilitate the acceptability and adoptability of the FBDR by the target groups.

## 5. Conclusions

Our results show that ensuring nutrient adequacy for 6–23-month-old Ethiopian children is difficult at least for some nutrients. Nutrient adequacy can be improved, in part, by promoting a diet with more vegetables (for >12 months children), legumes and animal source food that is currently part of the children’s diets. However, the results suggest that even if the FBDRs are fully adopted, intakes of some nutrients, in particular, zinc, iron and perhaps niacin might remain suboptimal for some children in the population and additional interventions are required. The best option to reduce the nutrient gaps is a combination of the regional FBDRs with MNPs (6 mg iron/serving) supplementation; daily (for children <12 months of age) and every other day (for children >12 months of age). Our findings confirm that providing MNPs may potentially improve the nutrient adequacy of the diets of these children, while not leading to substantial excessive intakes. It is important to emphasize that MNP should not replace the feeding recommendations, but should be promoted in addition to these FBDRs together with breastfeeding on demand during the first two years of age. Our findings further suggest that region-specific FBDR are required, to account for differences between regions in food availability and dietary habits and to increase the acceptability of the recommendations. Hence, targeted approaches and dosing instructions have to be considered separately for children below 12 months of age and children above 12 months of age. The study also confirms the usefulness of LP analysis in order to explore and evaluate the effect of different options for nutrition interventions so as to inform policymakers.

## Figures and Tables

**Table 1:** Nutrient composition of local complementary food per 100 g and different supplements per serving size, per region for young children in Ethiopia.

	CF * Tigray	CF * Amhara	CF * Oromia	CF * SNNPR	MNP **	Sq-LNS ***
	100 g	100 g	100 g	100 g	1 g	20 g
Energy (kcal)	351	357	371	373	0	118
Protein (g)	13.4	13.1	10.7	11.2	0	2.6
Water (g)	9.8	9	8.0	7.5	0	4
Fat (g)	1.7	2.1	2.8	2.9	0	9.6
Carbohydrate (g)	73.2	73.9	76.4	76.2	0	5.3
Calcium (mg)	57.5	42.8	38.8	27.7	0	280
Iron (mg)	11.9	7.8	10.8	4.7	6	6
Zinc (mg)	1.6	1.7	1.2	1.5	4.1	8
Vitamin C (mg)	0.5	1.4	2.6	3.2	30	30
Thiamin (mg)	0.3	0.3	0.3	0.3	0.5	0.3
Riboflavin (mg)	0.2	0.2	0.1	0.1	0.5	0.4
Niacin (mg)	3.3	2.9	1.5	1.6	6	4
Vitamin B6 (mg)	0.3	0.3	0.3	0.2	0.5	0.3
Folate (µg dietary eq.)	138.7	130	107.2	103.8	150	80
Vitamin B12 (mg)	0	0	0	0	0.9	0.5
Vitamin A (mg)	0.8	1.5	1.5	1.9	400	400

* CF = Local complementary Food product, daily portion sizes for CF were 40 g for 6–11 months old children and 60 g for 12–23-month old children, ** MNP = MicroNutrient Powder Supplement, *** Sq-LNS = Small quantity Lipid base Nutrient Supplement; SNNPR = South Nations, Nationalities and Peoples Region.

**Table 2:** Characteristics of study children by age group and region in four regions in Ethiopia.

Characteristics	Tigray	Amhara	Oromia	SNNPR
Total number	472	659	675	692
6–8 months	89	122 ‡	135	151
9–11 months	86	120	129	129
12–23 months	297	417 ‡	411 ‡	412
Sex-male n (%)				
6–8 months	39 (43.8)	68 (56.2)	73 (54.1)	73 (48.3)
9–11 months	38 (44.2)	57 (47.5)	77 (59.7)	67 (51.9)
12–23 months	135 (45.5)	216 (51.9)	236 (57.3)	227 (55.1)
Place of residence n (%)				
Urban	91 (19.3)	90 (13.7)	70 (10.4)	71 (10.3)
Rural	381 (80.7)	569 (86.3)	605 (89.6)	621 (89.7)
Nutritional status				
HAZ * (mean ± SD)	−1.73 ± 1.39	−1.61 ± 1.79	−1.21 ± 2.00	−1.37 ± 1.72
Stunting n (%)	201 (42.6)	267 (40.8) ‡‡	227 (33.7)	241 (34.8)
WAZ ** (mean ± SD)	−1.45 ± 1.09	−1.33 ± 1.24	−1.16 ± 1.40	−1.03 ± 1.34
Underweight n (%)	145 (30.8)	185 (28.2) ‡‡	172 (25.5)	144 (20.8)
WHZ *** (mean ± SD)	−0.73 ± 1.10	−0.66 ± 1.27	−0.69 ± 1.31	−0.40 ± 1.21
Wasting n (%)	54 (11.5)	76 (11.7) ‡‡	97 (14.4)	59 (8.5)

* HAZ-Height-for-Age Z Score, ** WAZ-Weight-for-Age Z Score, *** WHZ-Weight-for-height Z Score. Stunting defined as HAZ <−2 of the standard deviation (SD), underweight WAZ <−2 SD and wasting WHZ <−2 SD were determined using the WHO Anthro software version 3.2.2.; ‡‡ Missing 4 in stunting, 1 in underweight, 7 in wasting; ‡ 1 undefined.

**Table 3:** Summary of problem nutrients in young children’s diets that can be solved with food-based dietary recommendations and those persisting, by age group and region.

		Calcium	Thiamin	Niacin	Vit.B6	Folate	Vit.B12	Vit.A	Iron	Zinc
Tigray	6–8 mo.									
	9–11 mo.									
	12–23 mo.									
Amhara	6–8 mo.									
	9–11 mo.									
	12–23 mo.									
Oromia	6–8 mo.									
	9–11 mo.									
	12–23 mo.									
SNNPR	6–8 mo.									
	9–11 mo.									
	12–23 mo.									
*		Nutrient requirements that can be met but require changes consistent with FBDR								
**		Nutrient requirements cannot be met by any combination of local foods								

* These are partial problem nutrients being nutrients when the minimized %RNI <70% and the maximized RNI ≥ 100%. ** These are problem nutrients being nutrients for which it is difficult to ensure nutrient adequacy with local foods alone (the maximized RNI is <100%).

**Table 4:** Summary of food-based recommendations (in addition to breastmilk) for young Ethiopian children, for different age groups per region.

	Food Group	Foods ^2^	Age Group		
			6 to 8 mo. ^1^	9 to 11 mo. ^1^	12 to 23 mo. ^1^
			s/wk. ^3^	s/wk. ^3^	s/wk. ^3^
**Tigray**	Dairy	Milk	7	7	7
	FICFP ^4^		7	-	-
	Grains	Wheat, teff	4	7	14
	Vegetables	Vitamin C rich vegetables	-	7	3 to 4
	Legumes	Broad beans, vetch, (chick) peas	-	14	14
	Eggs		-	-	7
**Amhara**	Dairy	Milk	7	7	7
	FICFP		7	-	-
	Grains	Wheat, teff	-	14	14
	Vegetables ^5^	Tomato, onions	-	-	14
	Legumes	Broad beans, lentils	7	7	21
	Starchy Roots	Potato	-	7	7
**Oromia**	Dairy	Milk	3 to 4	3 to 4	3 to 4
	Grains	Wheat, teff	7	14	14
	Vegetables ^5^	Tomato, onion	-	14	14
	Legumes	Broad beans, lentils	7	3 to 4	14
	Starchy Roots	Potato	7	-	-
	Eggs		-	-	7
**SNNPR**	Dairy	(butter) milk	3 to 4	3 to 4	7
	Grains	Barley, millet, tef	14	35	21
	DGLV ^6^	Kale	-	7	-
	Legumes	Chickpeas, kidney beans	21	14	21
	Starchy roots	Potato	-	3 to 4	-
	Eggs		-	-	7

^1^ Months old; ^2^ Recommended foods within group; ^3^ Number of servings per week; ^4^ Fortified Infant Cereal Food Product; ^5^ Tomatoes and onion 14 servings in total; ^6^ Dark green leafy vegetables.

**Table 5:** Calculated inadequate and excess intake of selected nutrients at usual intake, addition of 1 MNP every other day (3.5 sachets/week) and daily, among Ethiopian children at different age groups.

Nutrient *(EAR)	Age Group 6–8 m (n = 495)		Age Group 9–11 m (n = 465)		Age Group 12–23 m (n = 1544)	
	Inadequate%	Excess%	Inadequate%	Excess%	Inadequate%	Excess%
Iron (10%) **						
Usual diet	77.7	4.6	67.1	3.7	40.1	18.7
+1/2MNP/d	67.3	5.3	52.8	4.1	26.2	20.5
+1MNP/d	39.8	6.1	26.6	4.5	10.4	22.2
Iron (5%) **						
Usual diet	86.5	4.6	81.6	3.7	52.9	18.7
+1/2MNP/d	82.3	5.3	75.3	4.1	46.4	20.5
+1MNP/d	75.8	6.1	66.5	4.5	35.5	22.2
Zinc (moderate bioavailability) ∞ (WHO cut-off)						
Usual diet	92.7	0	92.3	0	68.6	0.1
+1/2MNP/d	0	0	0	0	8.2	0.1
+1MNP/d	0	0.2	0	0	0	0.1
Zinc (low bioavailability) ∞ (WHO cut-off)						
Usual diet	98.6	0	100	0	96.1	0.1
+1/2MNP/d	96.0	0	97.2	0	87.3	0.1
+1MNP/d	0	0.2	0	0	53.9	0.1
Zinc ≡ (IZiNCG cut-off)						
Usual diet		0.4		0		2.4
+1/2MNP/d		1.6		0.4		6.9
+1MNP/d		21.2		51.0		22.4

* Analysis was made using square root transformation for iron and log10 transformation for zinc and data was back transformed to assess the inadequate and excess intake; ** EAR for iron: Based on IOM (10%bioavailability) 6.9 mg/d and IOM (5% bioavailability) 13.8 mg/d for 6–11 months (p. 324) [50] and based on WHO/FAO (10% of bioavailability) 5.8 mg/d and WHO/FAO (5% bioavailability) 11.6 mg/d for 12–23 months (p 148) [51]; ∞ EAR for Zinc (moderate bioavailability) 2.5 mg/d and (low bioavailability) 5.0 mg/d for 6–11 months based on IOM (p. 466) [50] and 3.4 mg/d (moderate bioavailability) and 6.9 mg/d (low bioavailability) for 12–23 months based on WHO/ FAO (p. 148) [51]; UL: Upper Level for Iron based on IOM 40 mg for all target groups (pp. 26–27) [50]. ∞ For zinc using WHO cut-off 13 mg for 6–11 months, and 23 mg for 12–23 months (p. S120) [52]; ≡ using IZiNCG cut-off 6 mg for 6–11 months, and 8 mg for 12–23 months (p. S120) [52]; MNP/d: Micronutrient Powder/day.

## Supplementary Materials

The following are available online at https://www.mdpi.com/2072-6643/11/6/1416/s1, Table S1. List of food groups and subgroups as defined by Optifood; Table S2. Reported intake and feeding practice by age group and region; Table S3. List of Foods consumed by >3% of children, median serving sizes (g/d), in Tigray, Amhara, Oromia, SNNP regions in Ethiopia; Tables S4–S15. The optimal, best-case scenario and worst-case scenario of the baseline diet, the worst-case scenario for the food based recommendations and each alternative combination expressed as a percentage of the recommended nutrient intake by age group and region.

## Abbreviations

CF: Complementary Food; EAR: Estimated Average Requirement; EHNRI: Ethiopian Health and Nutrition Research Institute; EPHI: Ethiopian Public Health Institute; FBDR: Food-Based Dietary Recommendations; FAO: Food and Agriculture Organization; FCT: Food Composition Table; HAZ: Height-for-Age Z score; IOM: Institute of Medicine; IYCF: Infant and Young Child Feeding; IZiNCG: International Zinc Nutrition Consultative Group; LP: Linear Programming; MNP: Micronutrient Powder; NRC: National Research Council; NFCS: National Food Consumption Survey; RNI: Recommended Nutrient Intake; SD: Standard Deviation; SNNPR: South Nations, Nationalities and Peoples Region; Sq-LNS: Small quantity Lipid-based Nutrient Supplement; UL: Tolerable Upper Intakes Level; UNICEF: United Nation Children’s Fund; WAZ: Weight-for-Age Z score; WHZ: Weight-for-Height Z score; WHO: World Health Organization.
