# Supplementary material for: Identifying Dietary Strategies to Improve Nutrient Adequacy among Ethiopian Infants and Young Children Using Linear Modelling
Source: Nutrients. 2019 Jun 24;11(6):1416. doi: 10.3390/nu11061416 (PMC6627485; doi:10.3390/nu11061416)
Supplement: Supplementary file 1 [file nutrients-11-01416-s001.pdf]

**Supplemental Table 1: List of food groups and subgroups as defined by Optifood software**

| | **Food group**/ sub-food group |
|---|---|
| **1** | **Added fats** |
| | Butter, ghee, margarine non-fortified |
| | Vegetable oil non-fortified |
| **2** | **Added sugars** |
| | Sugar non-fortified |
| **3** | **Bakery & breakfast cereals** |
| | Biscuits, sweet cookies |
| | Ready-to-eat cereals, fortified (commercial infant food) |
| **4** | **Dairy products** |
| | Fluid milk non-fortified |
| | Other dairy excluding butter |
| **5** | **Fruits** |
| | Other fruits |
| **6** | **Grains & grain products** |
| | Refined grains and products non-fortified |
| | Whole grains and products non-fortified |
| **7** | **Legumes, nuts & seeds** |
| | Cooked beans, lentils, peas |
| **8** | **Vegetables** |
| | Other vegetables |
| | Vitamin A source dark leafy greens |
| | Vitamin C rich vegetables |
| **9** | **Starchy roots and plants** |
| | Other starchy plant |
| **10** | **Meat, fish and eggs** |
| | Egg whole, raw |

**Supplemental Table 2. Reported intake and feeding practices by Ethiopian Children, by age group and region.**

| Age group | | Tigray | Amhara | Oromia | SNNPR | Average of all regions |
|---|---|---|---|---|---|---|
| 6-8 months | Breast milk % | 99 | 97 | 95 | 98 | 97 |
| | Foods consumed (n) | 74 | 82 | 93 | 70 | 80 |
| | Foods consumed by >3% of children(n) | 28 | 26 | 24 | 33 | 28 |
| 9-11months | Breast milk% | 95 | 93 | 89 | 94 | 93 |
| | Foods consumed (n) | 78 | 99 | 100 | 94 | 93 |
| | Foods consumed by >3% of children(n) | 29 | 35 | 40 | 47 | 38 |
| 12-23 months | Breast milk% | 86 | 90 | 85 | 91 | 88 |
| | Foods consumed (n) | 138 | 196 | 196 | 159 | 172 |
| | Foods consumed by >3% of children(n) | 48 | 52 | 53 | 56 | 52 |

**Online Supporting Material**

**Supplemental Table 3.**

## Table 3. List of Foods consumed by >3% of children, median serving sizes (g/d), in Tigray, Amhara, Oromia, SNNP regions in Ethiopia

| | Tigray | | | Amhara | | | Oromia | | | SNNPR | | |
|---|---|---|---|---|---|---|---|---|---|---|---|---|
| | Age of children in months | | | | | | | | | | | |
| | 6-8 | 9-11 | 12-23 | 6-8 | 9-11 | 12-23 | 6-8 | 9-11 | 12-23 | 6-8 | 9-11 | 12-23 |
| Added fats | | | | | | | | | | | | |
| Butter, spiced, clarified | - | - | - | - | - | - | 6 | 8 | 2 | 3 | 3 | 5 |
| Butter, un-spiced, raw | - | - | - | - | - | - | - | - | - | 7 | - | 8 |
| Oil, liquid | 1 | 3 | 4 | 1 | 2 | 3 | 3 | 4 | 4 | - | 1 | 2 |
| Shortening, fractionated, palm | 3 | 5 | 3 | - | 2 | 3 | 4 | 6 | 4 | - | 4 | 3 |
| Added sugars | | | | | | | | | | | | |
| Sugar, refined | 5 | 5 | 8 | 7 | 7 | 12 | 12 | 14 | 11 | 9 | 11 | 13 |
| Bakery and breakfast cereals | | | | | | | | | | | | |
| Biscuits, sweet cookies | - | - | - | - | - | - | 18 | 16 | - | - | - | - |
| Fortified infant cereal food | 7 | - | - | 4 | - | - | - | - | - | - | - | - |
| Dairy products | | | | | | | | | | | | |
| Buttermilk, cow | - | - | - | - | - | - | - | - | - | 307 | 154 | 173 |
| Milk, cow, boiled | - | - | - | 221 | 94 | 129 | 94 | 253 | 188 | - | 261 | 204 |
| Milk, cow, fresh | 52 | 137 | 126 | 134 | 162 | 129 | 218 | 267 | 234 | 148 | 113 | 173 |
| Sour milk, cow | - | - | - | - | - | - | - | - | - | - | 173 | 140 |
| Fruits | | | | | | | | | | | | |
| Avocado, fresh | - | - | - | - | - | - | - | - | - | - | - | 74 |
| Banana, fresh | - | - | - | - | - | - | - | - | - | - | - | 105 |
| Prickly pear | - | - | 139 | - | - | - | - | - | - | - | - | - |
| Grain and grain products | | | | | | | | | | | | |
| Barley black, flour | - | - | 16 | 1 | 3 | 3 | 14 | 16 | 5 | 1 | 3 | 3 |
| Barley white, flour | 9 | 13 | 26 | 2 | 9 | 6 | 9 | 18 | 10 | - | 16 | 5 |
| Corn yellow, flour | - | - | 5 | - | - | 2 | 3 | 2 | 5 | - | 3 | 4 |
| Corn white, flour | - | 4 | 14 | - | 24 | 13 | 5 | 21 | 47 | - | 21 | 39 |
| Emmer wheat, flour | - | - | - | 12 | 18 | 2 | - | 9 | 14 | - | 3 | 5 |
| Macaroni, pasta dry | 18 | 17 | 23 | - | 20 | 23 | - | 17 | 16 | - | - | - |
| Millet black, flour | - | - | 7 | - | - | 4 | - | - | 3 | 1 | 3 | 3 |
| Millet mixed, flour | - | - | 6 | - | - | 10 | 1 | - | 6 | 1 | 3 | 3 |
| Millet white, flour | - | - | 4 | - | - | 2 | - | - | - | - | 3 | 3 |
| Rice, wholegrain | - | - | 45 | - | - | - | - | - | - | - | - | - |
| Sorghum red, flour | 8 | 14 | 16 | - | 12 | 7 | 4 | 17 | 28 | 2 | 3 | 5 |
| Sorghum mixed, flour | 3 | 3 | 11 | 1 | 8 | 13 | 7 | 6 | 17 | - | 4 | 10 |
| Sorghum white, flour | 9 | 16 | 20 | 6 | 14 | 19 | 13 | 25 | 20 | - | 10 | 25 |
| Tef red, flour | 10 | 15 | 29 | 8 | 21 | 9 | 11 | 14 | 25 | 3 | 3 | 8 |
| Tef mixed, flour | 4 | 17 | 20 | 11 | 21 | 36 | 12 | 5 | 11 | 2 | 3 | 9 |
| Tef white, flour | 33 | 17 | 21 | 4 | 4 | 23 | 14 | 9 | 19 | 14 | 17 | 16 |
| Wheat black, flour | 5 | 20 | 69 | - | - | 10 | 4 | - | 3 | - | 3 | 3 |
| Wheat mixed, flour | - | 5 | 6 | 2 | 16 | 13 | 6 | 29 | 21 | - | 3 | 3 |

**Online Supporting Material**

| | | | | | | | | | | | | |
|---|---|---|---|---|---|---|---|---|---|---|---|---|
| Wheat white, flour | 15 | 31 | 39 | 14 | 10 | 21 | 12 | 29 | 40 | 1 | 8 | 25 |
| Wheat wholegrain, flour | - | - | - | - | 13 | 59 | - | 45 | 62 | - | - | 73 |
| Legumes, nuts and seeds | | | | | | | | | | | | |
| Broad beans, flour | 1 | - | 4 | 3 | 2 | 6 | - | 9 | 8 | 1 | 3 | 3 |
| Broad beans, split | - | - | 7 | - | - | 16 | - | - | 9 | - | - | - |
| Broad beans, whole fresh roasted | - | - | 8 | - | - | - | - | - | 10 | - | - | - |
| Chickpeas, flour | - | 12 | 3 | 1 | - | 4 | - | - | 4 | 1 | 3 | 3 |
| Kidney beans, whole, dried | - | - | - | - | - | 2 | - | - | - | 1 | 3 | 8 |
| Lentil, flour | - | - | - | 1 | - | 2 | - | - | - | 1 | 3 | 3 |
| Lentil, split | - | - | - | - | 4 | 12 | - | - | - | - | - | - |
| Lupine, raw | - | - | - | - | - | 2 | - | - | - | 1 | 3 | 3 |
| Partially ground grass pea | - | - | - | - | - | 9 | - | - | - | - | - | - |
| Peas, flour | 4 | 4 | 6 | 2 | - | 5 | 2 | 5 | 7 | 1 | 2 | 4 |
| Peas, split | - | - | 14 | - | - | 20 | - | - | - | - | - | - |
| Peas whole, fresh, roasted | 3 | 12 | 10 | - | - | - | - | 15 | 11 | - | - | - |
| Vetch, flour | 1 | 4 | 7 | 7 | 6 | 9 | - | 4 | 7 | 1 | 3 | 4 |
| Meat, fish and eggs | | | | | | | | | | | | |
| Egg whole, raw | - | 35 | 16 | - | 16 | - | 28 | 43 | 43 | - | 22 | 25 |
| Starchy roots and plants | | | | | | | | | | | | |
| False banana, flour | - | - | - | - | - | - | - | - | - | - | 39 | 48 |
| False banana, kocho | - | - | - | - | - | - | - | - | 69 | - | 70 | 55 |
| Potato Irish, boiled | - | - | - | - | - | 40 | - | - | 142 | - | 106 | 120 |
| Potato Irish, raw | - | - | - | - | 38 | 32 | 13 | 41 | 48 | - | 79 | 43 |
| Taro, boiled | - | - | - | - | - | - | - | - | - | - | - | 104 |
| Vegetables | | | | | | | | | | | | |
| Chili, boiled | - | - | - | - | 1 | 2 | - | - | - | - | - | - |
| Ethiopian kale, raw | - | - | - | - | - | - | - | - | 45 | - | 29 | 27 |
| Green pepper, raw | - | - | 1 | - | - | 7 | - | 4 | 6 | - | 11 | 4 |
| Onion bulb, raw or boiled | - | 2 | 8 | - | - | 9 | - | 9 | 12 | - | - | - |
| Onion shallot, raw | 4 | 6 | 7 | 10 | 4 | 15 | 13 | 12 | 9 | - | 4 | 5 |
| leek, raw | - | - | - | - | 8 | - | - | - | - | - | - | 4 |
| Tomato, raw | 8 | 7 | 12 | - | 6 | 18 | - | 18 | 15 | - | - | - |

**Supplemental Table 4-Table 15**

**Supplemental Table 4. The optimal[1], best-case scenario[2] and worst-case scenario[3] of the baseline diet, the worst-case scenario for the food based recommendations and each alternative combination expressed as a percentage of the recommended nutrient intake in 6 to 8 mo. infants of Tigray region.**

| | Protein | Fat | Calcium | Vit. C | Vit B-1 | Vit. B-2 | Niacin | Vit. B-6 | Folate | Vit. B-12 | Vit. A | Iron[4] | Zinc[5] | N[6] |
|---|---|---|---|---|---|---|---|---|---|---|---|---|---|---|
| Baseline diet[7] | 129.7 | 160.5 | 82.4 | 124.5 | 126.1 | 147.7 | 104.1 | 100 | 111.6 | 122.1 | 114.6 | 37.1 | 24.1 | 11 |
| best-case scenario[8] | 137.4 | 169.8 | 82.7 | 125.1 | 127.5 | 148.6 | 106.3 | 101.3 | 112.8 | 122.2 | 114.9 | 37.4 | 25.1 | 11 |
| worst-case scenario | 107.2 | 145.4 | 54.4 | 109.9 | 44.4 | 66.1 | 39.2 | 40.9 | 48.7 | 47.6 | 100.5 | 5.4 | 17.4 | 4 |
| FBDR[9] | 115.1 | 159.5 | 80.7 | 113.7 | 111.7 | 140.9 | 93.7 | 80.8 | 98.4 | 121.9 | 114 | 31.5 | 22.4 | 11 |
| CF 3.5 s/wk.[10] | 114.7 | 145.2 | 56 | 110.2 | 56.3 | 71.8 | 48.1 | 52.2 | 78.2 | 47.6 | 100.5 | 14.6 | 18.1 | 6 |
| CF 7 s/wk. | 134.7 | 145.8 | 58.5 | 110.5 | 74.8 | 80.6 | 61.8 | 69.7 | 110.6 | 47.7 | 100.6 | 26.7 | 21.1 | 7 |
| MNP 3.5 s/wk. | 107.2 | 145.4 | 54.4 | 159.9 | 127.7 | 128.6 | 114.2 | 124.2 | 142.5 | 111.9 | 100.5 | 21.5 | 41.8 | 10 |
| MNP 7 s/wk. | 107.2 | 145.4 | 54.4 | 209.9 | 211.1 | 191.1 | 189.2 | 207.6 | 236.2 | 176.2 | 100.5 | 37.6 | 66.2 | 10 |
| sq-LNS 3.5 s/wk. | 101.6 | 167.4 | 88.3 | 159.8 | 85.2 | 111.6 | 82.6 | 80.9 | 94.2 | 83.1 | 150.5 | 18.2 | 62.4 | 11 |
| sq-LNS 7 s/wk. | 104.3 | 189.9 | 122.7 | 209.8 | 130.9 | 159.4 | 129.2 | 124.3 | 141.2 | 118.6 | 200.5 | 33.3 | 108.6 | 12 |
| FBDR+ CF 2 s/wk. | 129.6 | 159.9 | 82.3 | 113.9 | 122.9 | 146.3 | 102.2 | 91.9 | 117.5 | 121.9 | 114 | 38.6 | 24.4 | 11 |
| FBDR+MNP 3.5 s/wk. | 115.1 | 159.5 | 80.7 | 163.7 | 194.9 | 203.4 | 168.7 | 164.1 | 192.2 | 186.2 | 114 | 47.7 | 46.8 | 11 |
| FBDR+ MNP 7 s/wk. | 115.1 | 159.5 | 80.7 | 213.7 | 278.3 | 265.9 | 243.7 | 247.4 | 285.9 | 250.5 | 114 | 63.8 | 71.2 | 12 |
| FBDR+ sq-LNS 2 s/wk. | 97.4 | 120.9 | 172.5 | 100.6 | 142.3 | 138.9 | 121.4 | 107.5 | 126.3 | 142.2 | 142.5 | 40.5 | 49.4 | 11 |
| FBDR+CF2 s/wk.+ MNP 3.5 s/wk. | 129.6 | 159.9 | 82.3 | 163.9 | 206.2 | 208.8 | 177.2 | 175.3 | 211.3 | 186.2 | 114 | 54.7 | 48.8 | 11 |

[1] The optimal diet formulated by using goal programming (Optifood module II)

[2] Each diet sequentially maximizes each micronutrient (Optifood module III)

[3] Each diet sequentially minimizes each micronutrient (Optifood module III)

[4] Recommended nutrient intake for iron assuming 5% absorption

[5] Recommended nutrient intake for zinc assuming low absorption.

[6] Number of nutrients to cover at least 70% of the recommended nutrient intake in the worst-case scenario.

[7] Nutritionally best possible diet (Optifood module II).

[8] Diet sequentially maximizes each nutrient (Optifood module III).

[9] Set of food based dietary recommendations selected.

[10] s/wk.=number of servings per week.

| | | | | | | | | | | | | | |
|---|---|---|---|---|---|---|---|---|---|---|---|---|---|
| FBDR+CF 2 s/wk. +MNP 7 s/wk.[1] | 129.6 | 159.9 | 82.3 | 213.9 | 289.5 | 271.3 | 252.2 | 258.6 | 305 | 250.5 | 114 | 70.9 | 73.2 | 13 |
| FBDR+ CF1 s/wk. + sq-LNS 1 s/wk. | 125.2 | 166.2 | 91.5 | 128.1 | 130.9 | 157.6 | 111.8 | 99.7 | 121.9 | 132.1 | 128.3 | 39.6 | 36.9 | 11 |

**Supplemental Table 5. The optimal[2], best-case scenario[3] and worst-case scenario[4] of the baseline diet, the worst-case scenario for the food based recommendation and alternative combinations expressed as a percentage of the recommended nutrient intake in 9 to 11 mo. infants of Tigray region.**

| | Protein | Fat | Calcium | Vit C | Vit B-1 | Vit B-2 | Niacin | Vit B-6 | Folate | Vit B-12 | Vit A | Iron[5] | Zinc[6] | N[7] |
|---|---|---|---|---|---|---|---|---|---|---|---|---|---|---|
| Baseline diet [8] | 177.3 | 153.5 | 75.9 | 121.8 | 100 | 134.5 | 57.6 | 105 | 148.6 | 122 | 132.7 | 71.9 | 33.2 | 11 |
| best-case scenario[9] | 194.3 | 187.9 | 92.9 | 130.1 | 108.5 | 174.9 | 71.1 | 119.1 | 157 | 164.6 | 139.6 | 74.1 | 35.7 | 12 |
| worst-case scenario | 112.5 | 124.3 | 52.3 | 102.5 | 59.8 | 68.3 | 38.9 | 55.4 | 53 | 44.8 | 93.8 | 11.3 | 18.6 | 4 |
| FBDR[10] | 153.6 | 156.1 | 88.9 | 127.5 | 82.3 | 145.8 | 47.2 | 80.4 | 125.6 | 115.6 | 106.2 | 60 | 29.5 | 10 |
| CF 3.5 s/wk.[11] | 118.1 | 123.6 | 53.5 | 102.9 | 67 | 72.4 | 50.5 | 64.2 | 81.7 | 44.8 | 93.8 | 19 | 19.2 | 6 |
| CF 7 s/wk. | 126.8 | 123.7 | 55.3 | 103.2 | 77.3 | 79 | 62.4 | 75.1 | 110.8 | 44.8 | 93.9 | 27.6 | 21.1 | 8 |
| MNP 3.5 s/wk. | 112.5 | 124.3 | 52.3 | 152.5 | 143.1 | 130.8 | 113.9 | 138.7 | 146.8 | 109.1 | 93.8 | 27.5 | 43.1 | 10 |
| MNP 7 s/wk. | 112.5 | 124.3 | 52.3 | 202.5 | 226.5 | 193.3 | 188.9 | 222 | 240.5 | 173.4 | 93.8 | 43.6 | 67.5 | 10 |
| sq-LNS 3.5 s/wk. | 104.9 | 143.8 | 85.9 | 152.5 | 97.1 | 112.6 | 84.9 | 93.6 | 98 | 80.3 | 143.8 | 23.1 | 63.5 | 11 |
| sq-LNS 7 s/wk. | 104.9 | 163.5 | 119.9 | 202.5 | 136.4 | 158.7 | 131.1 | 132.7 | 143 | 115.8 | 193.8 | 35.2 | 109.1 | 12 |
| FBDR+ CF 1 s/wk. | 161.2 | 156.3 | 89.7 | 127.6 | 88.5 | 148.8 | 51.9 | 86.8 | 135.5 | 115.6 | 106.2 | 63.7 | 30.6 | 10 |
| FBDR+MNP 3.5 s/wk. | 153.6 | 156.1 | 88.9 | 177.5 | 165.7 | 208.3 | 122.2 | 163.7 | 219.4 | 179.9 | 106.2 | 76.2 | 53.9 | 12 |
| FBDR+ MNP7 s/wk. | 153.6 | 156.1 | 88.9 | 227.5 | 249 | 270.8 | 197.2 | 247 | 313.1 | 244.2 | 106.2 | 92.3 | 78.3 | 13 |

[1]Best alternative option

[2] The optimal diet formulated by using goal programming (Optifood module II)

[3] Each diet sequentially maximizes each micronutrient (Optifood module III)

[4] Each diet sequentially minimizes each micronutrient (Optifood module III)

[5] Recommended intake for iron assuming 5% absorption.

[6] Recommended intake for zinc assuming low absorption.

[7] Number of nutrients that cover at least 70% of the recommended nutrient intake in the worst case scenario.

[8] Nutritionally best possible diet (Optifood module II)

[9] Diet sequentially maximizes each nutrient (Optifood module III).

[10] Set of food based dietary recommendations selected.

[11] s/wk.=number of servings per week.

[10] NP= Combination not possible due to energy constraints

| | | | | | | | | | | | | | | |
|---|---|---|---|---|---|---|---|---|---|---|---|---|---|---|
| FBDR+ MNP 6 s/wk.[1] | 153.6 | 156.1 | 88.9 | 213.2 | 225.2 | 252.9 | 175.8 | 223.2 | 286.3 | 225.8 | 106.2 | 87.7 | 71.4 | 13 |
| FBDR+ sq-LNS 1 s/wk. | 157.3 | 162 | 98.9 | 141.8 | 96.6 | 160 | 61.5 | 94.6 | 139.9 | 125.8 | 120.5 | 64.7 | 43.1 | 10 |
| FBDR+ CF1 s/wk. + MNP3.5 s/wk. | 188.2 | 170.2 | 89.4 | 170.8 | 167.9 | 225.6 | 121.4 | 171.1 | 241.9 | 228.6 | 139.1 | 75.1 | 54.5 | 12 |
| FBDR+ CF1 s/wk. + MNP 7 s/wk. | 188.2 | 170.2 | 89.4 | 220.8 | 251.2 | 288.1 | 196.4 | 254.4 | 335.7 | 292.9 | 139.1 | 91.2 | 78.9 | 13 |
| FBDR+ CF 1 s/wk. + MNP 6 s/wk. | 188.2 | 170.2 | 89.4 | 206.5 | 227.4 | 270.3 | 175 | 230.6 | 308.9 | 274.5 | 139.1 | 86.6 | 71.9 | 13 |
| FBDR+ CF+ sq-LNS | NP[2] | NP | NP | NP | NP | NP | NP | NP | NP | NP | NP | NP | NP | NP |

**Supplemental Table 6. The optimal[3], best-case scenario[4] and worst-case scenario[5] of the baseline diet; the worst-case scenario for the food based recommendations and alternative combinations expressed as a percentage of the recommended nutrient intake in 12 to 23 mo. infants of Tigray region.**

| | Protein | Fat | Calcium | Vit C | Vit B-1 | Vit B-2 | Niacin | Vit B-6 | Folate | Vit B-12 | Vit A | Iron[6] | Zinc[7] | N[8] |
|---|---|---|---|---|---|---|---|---|---|---|---|---|---|---|
| Baseline diet[9] | 212.3 | 121.6 | 100 | 177.6 | 95.7 | 146.4 | 59.4 | 108 | 81.7 | 100 | 111.2 | 100 | 64.8 | 11 |
| best-case scenario[10] | 223.2 | 137.8 | 109.9 | 193.9 | 117.3 | 161.4 | 86.1 | 127.7 | 87.1 | 101.4 | 111.8 | 144.3 | 70.4 | 13 |
| worst-case scenario | 143.4 | 90.5 | 40.9 | 91.4 | 59.5 | 61.1 | 37.1 | 65.8 | 35.7 | 31.9 | 83.6 | 39.9 | 24.4 | 4 |
| FBDR[11] | 213.4 | 119.5 | 70.6 | 114.8 | 99.8 | 140 | 70.4 | 89.8 | 74.5 | 99.9 | 110.2 | 86.3 | 45.7 | 12 |
| CF 3.5 s/wk.[12] | 152.1 | 89.5 | 41.6 | 91.9 | 62.1 | 66 | 45.2 | 67.65 | 57.45 | 31.9 | 83.7 | 57.25 | 24.25 | 4 |
| CF 7 s/wk. | 165.1 | 89.7 | 43.4 | 92.4 | 68.5 | 72.4 | 55.7 | 72 | 80.4 | 31.9 | 83.7 | 76.7 | 26.7 | 8 |
| MNP 3.5 s/wk. | 143.4 | 90.5 | 40.9 | 141.4 | 109.5 | 111.1 | 87.1 | 115.8 | 85.7 | 81.9 | 83.6 | 65.7 | 49.1 | 10 |
| MNP 7 s/wk. | 143.4 | 90.5 | 40.9 | 191.4 | 159.5 | 161.1 | 137.1 | 165.8 | 135.7 | 131.9 | 83.6 | 91.6 | 73.8 | 12 |
| sq-LNS 3.5 s/wk. | 136.1 | 105.6 | 67.3 | 141.4 | 79.6 | 96.1 | 65.7 | 85.1 | 58.5 | 59.4 | 133.6 | 57.7 | 69.1 | 7 |
| sq-LNS 7 s/wk. | 136.1 | 120.8 | 93.8 | 191.4 | 100.3 | 132.5 | 94.6 | 105 | 82.5 | 87 | 183.6 | 76.7 | 114.2 | 13 |

[1] Best alternative option

[2] NP= Combination not possible due to energy constraints

[3] The optimal diet formulated by using goal programming (Optifood module II)

[4] Each diet sequentially maximizes each micronutrient (Optifood module III)

[5] Each diet sequentially minimizes each micronutrient (Optifood module III)

[6] Recommended nutrient intake for iron assuming 5% absorption.

[7] Recommended nutrient intake for zinc assuming low absorption.

[8] Number of nutrients that cover at least 70% of the recommended nutrient intake in the worst-case scenario

[9] Nutritionally best possible diet (Optifood module II)

[10] Diet sequentially maximizes each nutrient (Optifood module III).

[11] Set of food based dietary recommendations selected.

[12] s/wk.=number of servings per week

| | | | | | | | | | | | | | | |
|---|---|---|---|---|---|---|---|---|---|---|---|---|---|---|
| FBDR+ CF | NP[1] | NP | NP | NP | NP | NP | NP | NP | NP | NP | NP | NP | NP | 0 |
| FBDR+ MNP 3.5 s/wk.[2] | 213.4 | 119.5 | 70.6 | 164.8 | 149.8 | 190 | 120.4 | 139.8 | 124.5 | 149.9 | 110.2 | 112.1 | 70.4 | 13 |
| FBDR+ MNP 7 s/wk. | 213.4 | 119.5 | 70.6 | 214.8 | 199.8 | 240 | 170.4 | 189.8 | 174.5 | 199.9 | 110.2 | 138 | 95.1 | 13 |
| FBDR+ sq-LNS 1 s/wk. | 216.8 | 124.2 | 78.6 | 129.1 | 108.3 | 151.4 | 79.9 | 98.3 | 82.1 | 107.8 | 124.5 | 93.7 | 59.4 | 12 |
| FBDR+CF +MNP | NP | NP | NP | NP | NP | NP | NP | NP | NP | NP | NP | NP | NP | 0 |
| FBDR+ CF+ sq-LNS | NP | NP | NP | NP | NP | NP | NP | NP | NP | NP | NP | NP | NP | 0 |

**Supplemental Table 7. The optimal,[3] best-case scenario[4] and worst-case scenario[5] of the baseline diet; the worst-case scenario for the food based recommendations and alternative combinations expressed as a percentage of the recommended nutrient intake in 6 to 8 mo. infants of Amhara region.**

| | Protein | Fat | Calcium | Vit. C | Vit B-1 | Vit B-2 | Niacin | Vit. B-6 | Folate | Vit. B-12 | Vit. A | Iron[6] | Zinc[7] | N[8] |
|---|---|---|---|---|---|---|---|---|---|---|---|---|---|---|
| Baseline[9] | 133.3 | 159.8 | 78 | 116.6 | 102.1 | 130.6 | 81 | 91.9 | 99.1 | 103.6 | 110.4 | 34 | 21.4 | 11 |
| best-case scenario[10] | 150.4 | 189.7 | 97.8 | 123.6 | 103.8 | 173.9 | 81.5 | 93.2 | 99.2 | 157 | 119.2 | 34.6 | 24.1 | 11 |
| worst-case scenario | 110.1 | 147.4 | 61.3 | 111.1 | 51.5 | 82.5 | 36.7 | 45.4 | 50.8 | 55.1 | 101.8 | 9.1 | 17.2 | 5 |
| FBDR[11] | 148 | 184.3 | 96.7 | 121 | 91.3 | 172.3 | 71.1 | 70.2 | 89 | 156.7 | 118.9 | 21.6 | 22.8 | 11 |
| CF 3.5 s/wk.[12] | 114.1 | 144.9 | 55.5 | 110.8 | 58.3 | 72.6 | 46.6 | 52.5 | 77.2 | 47.6 | 100.6 | 12.3 | 18 | 6 |

[1] NP= Combination not possible due to energy constraints

[2] Best alternative option

[3] The optimal diet formulated by using goal programming (Optifood module II)

[4] Each diet sequentially maximizes each micronutrient (Optifood module III)

[5] Each diet sequentially maximizes each micronutrient (Optifood module III)

[6] Recommended nutrient intake for iron assuming 5% absorption

[7] Recommended nutrient intake for zinc assuming low absorption

[8] Number of nutrients to cover at least 70% of the recommended nutrient intake in the worst-case scenario.

[9] Nutritionally best possible diet (Optifood module II)

[10] Diet sequentially maximizes each nutrient (Optifood module III).

[11] Set of food based dietary recommendations selected

[12] s/wk.=number of servings per week

**Online Supporting Material**

| | | | | | | | | | | | | | | |
|---|---|---|---|---|---|---|---|---|---|---|---|---|---|---|
| CF 7 s/wk. | 133 | 145.8 | 57 | 111.7 | 70.3 | 78.1 | 58.7 | 65.5 | 106.2 | 47.7 | 100.6 | 17.8 | 21.7 | 7 |
| MNP 3.5 s/wk. | 110.1 | 147.4 | 61.3 | 161.1 | 134.8 | 145 | 111.7 | 128.8 | 144.6 | 119.4 | 101.8 | 25.5 | 41.6 | 10 |
| MNP 7 s/wk. | 110.1 | 147.4 | 61.3 | 211.1 | 218.1 | 207.5 | 186.7 | 212.1 | 238.3 | 183.7 | 101.8 | 41.4 | 66 | 10 |
| sq-LNS 3.5 s/wk. | 102.1 | 166.8 | 88.6 | 159.9 | 90.8 | 115.7 | 82.7 | 84.2 | 95.8 | 83.1 | 150.5 | 20.9 | 61.7 | 11 |
| sq-LNS 7 s/wk. | 103.9 | 188.9 | 122.7 | 209.8 | 131 | 159.5 | 129.2 | 124.4 | 141.2 | 118.6 | 200.5 | 33.3 | 108.6 | 12 |
| FBDR+ CF | NP[1] | NP | NP | NP | NP | NP | NP | NP | NP | NP | NP | NP | NP | 0 |
| FBDR+ MNP 3.5 s/wk. | 148 | 184.3 | 96.7 | 121 | 91.3 | 172.3 | 71.1 | 70.2 | 89 | 156.7 | 118.9 | 21.6 | 22.8 | 11 |
| *FBDR+ MNP 7* s/wk.[2] | 148 | 184.3 | 96.7 | 221 | 257.9 | 297.3 | 221.1 | 236.9 | 276.5 | 285.3 | 118.9 | 53.8 | 71.6 | 12 |
| FBDR+ sq-LNS | NP | NP | NP | NP | NP | NP | NP | NP | NP | NP | NP | NP | NP | 0 |
| FBDR+CF +MNP | NP | NP | NP | NP | NP | NP | NP | NP | NP | NP | NP | NP | NP | 0 |
| FBDR+ CF+ sq-LNS | NP | NP | NP | NP | NP | NP | NP | NP | NP | NP | NP | NP | NP | 0 |

[1] NP= Combination not possible due to energy constraints

[2] Best alternative option

**Supplemental Table 8. The optimal[1], best-case scenario[2] and worst-case scenario[3] of the baseline diet; the worst-case scenario for the food based recommendations and alternative combinations expressed as a percentage of the recommended nutrient intake for 9 to 11 mo. infants of Amhara region.**

| | Protein | Fat | Calcium | Vit. C | Vit B-1 | Vit B-2 | Niacin | Vit. B-6 | Folate | Vit. B-12 | Vit. A | Iron[4] | Zinc[5] | N[6] |
|---|---|---|---|---|---|---|---|---|---|---|---|---|---|---|
| Baseline[7] | 152.4 | 145.3 | 74.4 | 130.1 | 98.4 | 132.6 | 68.4 | 128.6 | 100 | 100 | 114.8 | 51.7 | 26.9 | 10 |
| best-case scenario[8] | 179 | 171.6 | 90.2 | 138.5 | 107.8 | 164.4 | 76.5 | 140.2 | 108.3 | 136.4 | 120.9 | 54.5 | 32.9 | 11 |
| worst-case scenario | 104.1 | 122.9 | 51.6 | 102.5 | 59.7 | 68.3 | 42 | 55.4 | 53 | 44.8 | 93.8 | 11.3 | 18.3 | 4 |
| FBDR[9] | 144.6 | 153.6 | 86.8 | 129.3 | 91.1 | 153.4 | 62.9 | 117.3 | 82 | 113.8 | 104.8 | 42.8 | 24.7 | 10 |
| CF 3.5 s/wk.[10] | 115.5 | 123.3 | 53 | 103.7 | 66.7 | 72.1 | 52.8 | 64.2 | 86.1 | 44.9 | 93.9 | 15.2 | 19.7 | 6 |
| CF 7 s/wk. | 131.4 | 124 | 54.7 | 104.9 | 80.4 | 80 | 65.3 | 77.5 | 120.7 | 44.9 | 93.9 | 22.3 | 22.9 | 8 |
| MNP 3.5 s/wk. | 104.1 | 122.9 | 51.6 | 152.5 | 143 | 130.8 | 117 | 138.7 | 146.7 | 109.1 | 93.8 | 27.4 | 42.7 | 10 |
| MNP 7 s/wk. | 104.1 | 122.9 | 51.6 | 202.5 | 226.4 | 193.3 | 192 | 222.1 | 240.5 | 173.4 | 93.8 | 43.5 | 67.1 | 10 |
| sq-LNS 3.5 s/wk. | 102.4 | 142.7 | 85.7 | 152.5 | 97.4 | 112 | 86.4 | 94.2 | 98 | 80.3 | 143.8 | 23.1 | 63.2 | 11 |
| sq-LNS 7 s/wk. | 102.4 | 162.8 | 119.9 | 202.5 | 136.7 | 159.1 | 132.4 | 133.6 | 143.1 | 115.8 | 193.8 | 35.2 | 108.8 | 12 |
| FBDR+ CF | NP[11] | NP | NP | NP | NP | NP | NP | NP | NP | NP | NP | NP | NP | 0 |
| FBDR+ MNP 3.5 s/wk. | 144.6 | 153.6 | 86.8 | 179.3 | 174.4 | 215.9 | 137.9 | 200.7 | 175.7 | 178.1 | 104.8 | 59 | 49.1 | 11 |
| *FBDR+ MNP 7* s/wk.[12] | 144.6 | 153.6 | 86.8 | 229.3 | 257.7 | 278.4 | 212.9 | 284 | 269.5 | 242.4 | 104.8 | 75.1 | 73.5 | 13 |
| FBDR+ sq-LNS | NP | NP | NP | NP | NP | NP | NP | NP | NP | NP | NP | NP | NP | 0 |
| FBDR+CF +MNP | NP | NP | NP | NP | NP | NP | NP | NP | NP | NP | NP | NP | NP | 0 |
| FBDR+ CF+ sq-LNS | NP | NP | NP | NP | NP | NP | NP | NP | NP | NP | NP | NP | NP | 0 |

1 The optimal diet formulated by using goal programming (Optifood module II).

2 Each diet sequentially maximizes each micronutrient (Optifood module III).

3 Each diet sequentially minimizes each micronutrient (Optifood module III).

4 Recommended nutrient intake for iron assuming a 5% absorption

5 Recommended nutrient intake for zinc assuming low absorption

6 Number of nutrient to cover at least 70% of the recommended nutrient intake in the worst-case scenario.

7 Nutritionally best possible diet (Optifood module II)

8 Diet sequentially maximizes each nutrient (Optifood module III).

9 Set of food based dietary recommendation selected

10 s/wk=number of Servings per week

11 NP= Combination not possible due to energy constraints

12 Best alternative option

**Supplemental Table 9. The optimal[1], best-case scenario[2] and worst-case scenario[3] of the baseline diet; the worst-case scenario for the food based recommendations and alternative combinations expressed as a percentage of the recommended nutrient intake for 12 to 23 mo. children from Amhara region.**

| | Protein | Fat | Calcium | Vit. C | Vit B-1 | Vit B-2 | Niacin | Vit. B-6 | Folate | Vit. B-12 | Vit. A | Iron[4] | Zinc[5] | N[6] |
|---|---|---|---|---|---|---|---|---|---|---|---|---|---|---|
| Baseline[7] | 214.5 | 117.2 | 77.7 | 135.3 | 98.5 | 145.6 | 67.8 | 111.5 | 99.1 | 84.2 | 95.6 | 168.1 | 37.1 | 11 |
| best-case scenario[8] | 224.4 | 130.7 | 78.4 | 141.2 | 106.1 | 148.9 | 71.8 | 125.5 | 99.8 | 84.3 | 95.7 | 173.9 | 42.4 | 12 |
| worst-case scenario | 151.9 | 91.1 | 45.8 | 94.3 | 65.1 | 72.9 | 41.6 | 64.6 | 40.3 | 31.9 | 83.6 | 45.7 | 24.1 | 5 |
| FBDR[9] | 192.6 | 113.7 | 74.1 | 134.2 | 82.3 | 136.9 | 60.4 | 101 | 88.1 | 84.2 | 95.5 | 143.7 | 29.2 | 11 |
| CF 3.5 s/wk.[10] | 147.5 | 90.2 | 41.7 | 93.1 | 63.2 | 68.1 | 47.4 | 62.5 | 56.4 | 31.9 | 83.7 | 48.3 | 24.2 | 4 |
| CF 7 s/wk. | 159.2 | 90.3 | 41.9 | 94.3 | 65.6 | 69.6 | 54.2 | 66.3 | 77.5 | 31.9 | 83.8 | 54.9 | 26.1 | 6 |
| MNP 3.5 s/wk. | 151.9 | 91.1 | 45.8 | 144.3 | 115.1 | 122.9 | 92.6 | 114.6 | 90.3 | 81.9 | 83.6 | 71.6 | 48.8 | 11 |
| MNP 7 s/wk. | 151.9 | 91.1 | 45.8 | 194.3 | 165.1 | 172.9 | 141.6 | 164.6 | 140.3 | 131.9 | 83.6 | 97.4 | 73.5 | 12 |
| sq-LNS 3.5 s/wk. | 132.1 | 106 | 69.1 | 142.7 | 83.7 | 104.2 | 70 | 83.2 | 60.4 | 59.4 | 133.6 | 61 | 68.8 | 8 |
| sq-LNS 7 s/wk. | 132.1 | 121.2 | 95 | 191.6 | 103.7 | 136 | 98.4 | 102.2 | 83.2 | 87 | 183.6 | 78.3 | 113.7 | 13 |
| FBDR+ CF 2 s/wk. | 213.4 | 114.6 | 75.5 | 135 | 92.2 | 143.1 | 68.8 | 111.4 | 103 | 84.2 | 95.5 | 155.2 | 32.8 | 11 |
| FBDR+ MNP 3.5 s/wk. | 192.6 | 113.7 | 74.1 | 184.2 | 132.3 | 186.9 | 110.4 | 151 | 138.1 | 134.2 | 95.5 | 169.6 | 53.9 | 12 |
| FBDR+MNP 7 s/wk. | 192.6 | 113.7 | 74.1 | 234.2 | 182.3 | 236.9 | 160.4 | 201 | 188.1 | 184.2 | 95.5 | 195.4 | 78.6 | 13 |
| FBDR+ MNP 6 s/wk. | 192.6 | 113.7 | 74.1 | 219.9 | 168 | 222.6 | 146.1 | 186.7 | 173.8 | 169.9 | 95.5 | 188 | 71.5 | 13 |
| *FBDR+ sq-LNS 3* s/wk.[11] | 202.9 | 127.7 | 98.1 | 177.1 | 107.8 | 171 | 88.9 | 126.5 | 111 | 107.8 | 138.3 | 165.9 | 70.5 | 13 |
| FBDR+CF 2 s/wk. +MNP 3.5 s/wk. | 213.4 | 114.6 | 75.5 | 185 | 142.2 | 193.1 | 118.8 | 161.4 | 153 | 134.2 | 95.5 | 181.1 | 57.5 | 12 |

[1] The optimal diet formulated by using goal programming (Optifood module II).

[2] Each diet sequentially maximizes each micronutrient (Optifood module III)

[3] Each diet sequentially minimizes each micronutrient (Optifood module III)

[4] Recommended nutrient intake for iron assuming 5% absorption

[5] Recommended nutrient intake for zinc assuming low absorption

[6] Number of nutrients to cover at least 70% of the recommended nutrient intake of the worst-case scenario.

[7] Nutritionally best possible diet (Optifood module II)

[8] Diet sequentially maximizes each nutrient (Optifood module III).

[9] Set of food based dietary recommendations selected

[10] s/wk=number of servings per week

[11] Best alternative option

**Online Supporting Material**

| | | | | | | | | | | | | | | |
|---|---|---|---|---|---|---|---|---|---|---|---|---|---|---|
| FBDR+CF 2 s/wk. +MNP 7 s/wk. | 213.4 | 114.6 | 75.5 | 235 | 192.2 | 243.1 | 168.8 | 211.4 | 203 | 184.2 | 95.5 | 206.9 | 82.2 | 13 |
| FBDR+CF 2 s/wk. +MNP 6 s/wk. | 213.4 | 114.6 | 75.5 | 220.7 | 177.9 | 228.8 | 154.5 | 197.1 | 188.7 | 169.9 | 95.5 | 199.5 | 75.1 | 13 |
| FBDR+ CF1 s/wk. + sq-LNS 2 | 209.9 | 123.5 | 90.8 | 163.2 | 104.3 | 162.7 | 83.6 | 123.2 | 110.8 | 100 | 124.1 | 164.2 | 58.6 | 13 |

**Supplemental Table 10. The optimal[1], best-case[2] and worst-case scenario[3] of the baseline diet; the worst-case scenario for the food based recommendations and alternative combinations expressed as a percentage of the recommended nutrient intake in children from 6 to 8 mo. of Oromia region.**

| | Protein | Fat | Calcium | Vit. C | Vit B-1 | Vit B-2 | Niacin | Vit. B-6 | Folate | Vit. B-12 | Vit. A | Iron[4] | Zinc[5] | N[6] |
|---|---|---|---|---|---|---|---|---|---|---|---|---|---|---|
| Baseline[7] | 144.6 | 164.3 | 69.1 | 125.3 | 74.5 | 105.8 | 45.9 | 100 | 90.2 | 100 | 129.3 | 38.4 | 23.3 | 9 |
| best-case scenario[8] | 168 | 205.3 | 97.8 | 134.3 | 81 | 165.5 | 61 | 109.2 | 94.3 | 161.5 | 139.3 | 45.2 | 28.8 | 10 |
| worst-case scenario | 83.8 | 143.7 | 53.2 | 109.9 | 34.9 | 62.1 | 31.6 | 29.3 | 44.3 | 47.6 | 100.5 | 2.3 | 13.8 | 4 |
| FBDR[9] | 130 | 167.3 | 78.4 | 126.8 | 67.9 | 118.3 | 46.5 | 77.7 | 67.3 | 95 | 108.1 | 30.5 | 21.2 | 8 |
| CF3.5 s/wk.[10] | 98.9 | 144.6 | 54.7 | 111.6 | 51.2 | 65.5 | 36.6 | 42.3 | 68 | 47.6 | 100.6 | 12.6 | 16.2 | 4 |
| CF 7 s/wk. | 122.6 | 147.1 | 56.6 | 113.3 | 71.3 | 71.3 | 44 | 59.8 | 94.8 | 47.6 | 100.7 | 24.2 | 19.2 | 7 |
| MNP 3.5 s/wk. | 83.8 | 143.7 | 53.2 | 159.9 | 118.3 | 124.6 | 106.6 | 112.7 | 138.1 | 111.9 | 100.5 | 18.5 | 38.2 | 10 |
| MNP 7 s/wk. | 83.8 | 143.7 | 53.2 | 209.9 | 201.6 | 187.1 | 181.6 | 196 | 231.8 | 176.2 | 100.5 | 34.6 | 62.6 | 10 |
| sq-LNS 3.5 s/wk. | 89.6 | 165.7 | 87.8 | 159.8 | 81.1 | 109.6 | 79.2 | 74.6 | 91.4 | 83.1 | 150.5 | 17.2 | 61 | 11 |
| sq-LNS 7 s/wk. | 103.9 | 188.8 | 122.7 | 209.8 | 130.8 | 159.4 | 129.2 | 124.2 | 141.2 | 118.6 | 200.5 | 33.3 | 108.6 | 12 |
| FBDR+ CF | NP[11] | NP | NP | NP | NP | NP | NP | NP | NP | NP | NP | NP | NP | 0 |
| FBDR+ MNP 3.5 s/wk. | 130.2 | 167.3 | 78.5 | 176.8 | 151.6 | 180.5 | 121.6 | 161.2 | 161.2 | 159.3 | 108.1 | 46.3 | 46.1 | 11 |
| FBDR+ MNP 7 s/wk.[12] | 130.2 | 167.3 | 78.5 | 226.8 | 234.9 | 243 | 196.6 | 244.5 | 254.9 | 223.5 | 108.1 | 62.4 | 64.8 | 11 |
| FBDR+ sq-LNS | NP | NP | NP | NP | NP | NP | NP | NP | NP | NP | NP | NP | NP | 0 |
| FBDR+CF +MNP | NP | NP | NP | NP | NP | NP | NP | NP | NP | NP | NP | NP | NP | 0 |
| FBDR+ CF+ sq-LNS | NP | NP | NP | NP | NP | NP | NP | NP | NP | NP | NP | NP | NP | 0 |

[1] The optimal diet formulated by using goal programming (Optifood module II).

[2] Each diet sequentially maximizes each micronutrient (Optifood module III).

[3] Each diet sequentially minimizes each micronutrient (Optifood module III).

[4] Recommended nutrient for iron intake assuming 5% absorption

[5] recommended nutrient intake for zinc assuming low absorption

[6] Number of nutrient to cover at least 70% of the recommended nutrient intake in the worst-case scenario.

[7] Nutritionally best possible diet (Optifood module II)

[8] Diet sequentially maximizes each nutrient (Optifood module III).

[9] Set of food based dietary recommendations selected

[10] s/wk=number of servings per week

[11] NP= Combination not possible due to energy constraints

[12] Best alternative option

**Supplemental Table 11. The optimal[1], best-case scenario[2] and worst-case scenario[3] of the baseline diet; the worst-case scenario for the food based recommendations and alternative combinations expressed as a percentage of the recommended nutrient intake for infants of 9 to 11 mo. in Oromia region.**

| | Protein | Fat | Calcium | Vit. C | Vit B-1 | Vit B-2 | Niacin | Vit. B-6 | Folate | Vit. B-12 | Vit. A | Iron[4] | Zinc[5] | N[6] |
|---|---|---|---|---|---|---|---|---|---|---|---|---|---|---|
| Baseline[7] | 181.8 | 161 | 80.3 | 133.3 | 100 | 155.1 | 65.8 | 111.6 | 105.8 | 146.2 | 142.8 | 44.8 | 35.2 | 10 |
| best-case scenario[8] | 202 | 218 | 113.7 | 167.7 | 114.2 | 221.5 | 99.5 | 166.8 | 133.6 | 232.7 | 155.8 | 51 | 37.9 | 11 |
| worst-case scenario | 75.8 | 122 | 49.9 | 102.5 | 36.2 | 59.6 | 31.6 | 33.9 | 43.6 | 44.8 | 93.8 | 2.8 | 14.2 | 4 |
| FBDR[9] | 148.2 | 154 | 86.5 | 131.3 | 87.9 | 147.6 | 63.5 | 91 | 79.8 | 113 | 105.8 | 31 | 29.9 | 10 |
| CF3.5 s/wk.[10] | 86.2 | 123 | 51.3 | 104.2 | 50.5 | 62.4 | 35.2 | 42.5 | 66 | 44.8 | 93.9 | 12.8 | 15.4 | 4 |
| CF 7 s/wk. | 105.8 | 124 | 53.1 | 105.9 | 69.4 | 67.5 | 42 | 58.5 | 92.1 | 44.8 | 94 | 24.1 | 18.2 | 5 |
| MNP 3.5 s/wk. | 75.8 | 122 | 49.9 | 152.5 | 119.5 | 122.1 | 106.6 | 117.3 | 137.3 | 109.1 | 93.8 | 18.9 | 38.6 | 10 |
| MNP 7 s/wk. | 75.8 | 122 | 49.9 | 202.5 | 202.9 | 184.6 | 181.6 | 200.6 | 231.1 | 173.4 | 93.8 | 35.1 | 63 | 10 |
| sq-LNS 3.5 s/wk. | 79.6 | 142 | 84.4 | 152.5 | 81.4 | 107 | 78.5 | 76.4 | 89.9 | 80.3 | 143.8 | 17.6 | 60.4 | 11 |
| sq-LNS 7 s/wk. | 89 | 162 | 119.3 | 202.5 | 129.1 | 155.7 | 127.3 | 123 | 138.6 | 115.8 | 193.8 | 33.3 | 107.7 | 12 |
| FBDR+ CF 1 s/wk. | 154.3 | 154 | 87 | 131.8 | 93.7 | 149.3 | 65.6 | 96.1 | 87.4 | 113 | 105.8 | 34.3 | 30.7 | 10 |

[1] The optimal diet formulated by using goal programming (Optifood module II).

[2] Each diet sequentially maximizes each micronutrient (Optifood module III).

[3] Each diet sequentially minimizes each micronutrient (Optifood module III).

[4] Recommended nutrient intake for iron assuming 5% absorption

[5] Recommended nutrient intake for zinc assuming low absorption

[6] Number of nutrient to cover at least 70% of the recommended nutrient intake in the worst-case scenario.

[7] Nutritionally best possible diet (Optifood module II)

[8] Diet sequentially maximizes each nutrient (Optifood module III).

[9] Set of food based dietary recommendations selected

[10] s/wk=number of servings per week

| | | | | | | | | | | | | | | |
|---|---|---|---|---|---|---|---|---|---|---|---|---|---|---|
| FBDR+ MNP 3.5 s/wk. | 148.2 | 153 | 86.5 | 181.3 | 171.2 | 210.1 | 138.5 | 174.3 | 173.5 | 177.3 | 105.8 | 47.1 | 54.3 | 11 |
| FBDR+ MNP 7 s/wk. | 148.2 | 153 | 86.5 | 231.3 | 254.5 | 272.6 | 213.5 | 257.7 | 267.3 | 241.6 | 105.8 | 63.2 | 78.7 | 12 |
| FBDR+ sq-LNS 1 s/wk. | 151.9 | 159 | 96.5 | 145.6 | 102.2 | 161.8 | 77.7 | 105.3 | 94.1 | 123.3 | 120 | 35.6 | 43.5 | 11 |
| FBDR+CF1 s/wk. +MNP 3.5 s/wk. | 154.3 | 154 | 87 | 181.8 | 177 | 211.8 | 140.6 | 179.4 | 181.2 | 177.3 | 105.8 | 50.4 | 55.1 | 11 |
| FBDR+CF 1 s/wk. +MNP 7 s/wk.[1] | 154.3 | 154 | 87 | 231.8 | 260.3 | 274.3 | 215.6 | 262.7 | 274.9 | 241.6 | 105.8 | 66.5 | 79.5 | 12 |
| FBDR+ CF+ sq-LNS | NP[2] | NP | NP | NP | NP | NP | NP | NP | NP | NP | NP | NP | NP | 0 |

**Supplemental Table 12. The optimal[3], best-case scenario[4] and worst-case scenario[5] of the baseline diet; the worst-case scenario for the food based recommendations and alternative combinations expressed as a percentage of the recommended nutrient intake in 12 to 23 mo. children of Oromia region.**

| | Protein | Fat | Calcium | Vit. C | Vit B-1 | Vit B-2 | Niacin | Vit. B-6 | Folate | Vit. B-12 | Vit. A | Iron[6] | Zinc[7] | N[8] |
|---|---|---|---|---|---|---|---|---|---|---|---|---|---|---|
| Baseline[9] | 248.5 | 130.5 | 100 | 159.8 | 103.4 | 185.5 | 75.1 | 120 | 100 | 131.4 | 223.4 | 134.9 | 46.9 | 12 |
| best-case scenario[10] | 265.2 | 170.3 | 118.3 | 192 | 115.5 | 225.8 | 95.5 | 162.8 | 104.5 | 171.4 | 231.5 | 162.3 | 53.4 | 12 |
| worst-case scenario | 120.7 | 90.7 | 38.8 | 91.4 | 56.5 | 58.2 | 32.7 | 59.7 | 34.3 | 31.8 | 83.6 | 42.4 | 22.2 | 3 |
| FBDR[11] | 239.3 | 125.4 | 92.6 | 152 | 99.6 | 174.2 | 76.1 | 112.6 | 96.7 | 120.8 | 221.1 | 120.5 | 42.6 | 12 |
| CF3.5 s/wk.[12] | 126 | 90.8 | 39.6 | 94 | 58 | 57 | 34.3 | 56.5 | 50.7 | 31.8 | 83.7 | 55 | 21.3 | 4 |
| CF 7 s/wk. | 132.7 | 91.4 | 40.8 | 96.5 | 64.1 | 59.9 | 36.7 | 59.9 | 67.2 | 31.8 | 83.8 | 69.8 | 22.5 | 4 |
| MNP 3.5 s/wk. | 120.7 | 90.7 | 38.8 | 141.4 | 106.5 | 108.2 | 82.7 | 109.7 | 84.3 | 81.8 | 83.6 | 68.2 | 46.9 | 10 |

[1] Best alternative option

[2] NP= Combination not possible due to energy constraints

[3] The optimal diet formulated by using goal programming (Optifood module II).

[4] Each diet sequentially maximizes each micronutrient (Optifood module III)

[5] Each diet sequentially minimizes each micronutrient (Optifood module III)

[6] Recommended nutrient intake for iron assuming 5% absorption

[7] Recommended nutrient intake for zinc assuming low absorption

[8] Number of nutrients that cover at least 70% of the recommended nutrient intake in the worst-case scenario.

[9] Nutritionally best possible diet (Optifood module II)

[10] Diet sequentially maximizes each nutrient (Optifood module III).

[11] Set of food based dietary recommendations selected

[12] Number of servings per week

| | | | | | | | | | | | | | | |
|---|---|---|---|---|---|---|---|---|---|---|---|---|---|---|
| MNP 7 s/wk. | 120.7 | 90.7 | 38.8 | 191.4 | 156.5 | 158.2 | 132.7 | 159.7 | 134.3 | 131.8 | 83.6 | 94.1 | 71.6 | 12 |
| sq-LNS 3.5 s/wk. | 118.8 | 105.9 | 65.9 | 141.4 | 77.5 | 92.7 | 62.6 | 78.7 | 58.3 | 59.4 | 133.6 | 60 | 67.3 | 7 |
| sq-LNS 7 s/wk. | 118.8 | 121.2 | 93.2 | 191.4 | 99 | 129.4 | 93.3 | 99.7 | 82.3 | 87 | 183.6 | 78.2 | 113 | 13 |
| FBDR+ CF 1 s/wk. | 244.4 | 126.1 | 93.7 | 152.7 | 103.5 | 173.9 | 75.1 | 114.7 | 102.4 | 120.8 | 221.1 | 131.2 | 44.6 | 12 |
| FBDR+MNP3.5 s/wk. | 239.3 | 125.4 | 92.6 | 202 | 149.6 | 224.2 | 126.1 | 162.6 | 146.7 | 170.8 | 221.1 | 146.4 | 67.3 | 12 |
| FBDR+MNP 7 s/wk. | 239.3 | 125.4 | 92.6 | 252 | 199.6 | 274.2 | 176.1 | 212.6 | 196.7 | 220.8 | 221.1 | 172.3 | 92 | 13 |
| FBDR+ MNP 4 s/wk.[1] | 239.3 | 125.4 | 92.6 | 209.1 | 156.8 | 231.3 | 133.3 | 169.8 | 153.8 | 177.9 | 221.2 | 150.1 | 70.8 | 13 |
| FBDR+ sq-LNS 1 s/wk. | 242.8 | 130.2 | 100.6 | 166.3 | 108.2 | 185.6 | 85.7 | 121.2 | 104.3 | 128.7 | 235.4 | 127.9 | 56.4 | 12 |
| FBDR+CF1 s/wk. +MNP 3.5 s/wk. | 242.1 | 126.8 | 92.9 | 202.7 | 151.3 | 223.3 | 124.2 | 162.9 | 151.2 | 170.8 | 221.1 | 151.5 | 68.1 | 12 |
| FBDR+CF1 s/wk. +MNP7 s/wk. | 242.1 | 125.8 | 92.9 | 252.7 | 201.3 | 273.3 | 174.2 | 212.9 | 201.2 | 220.8 | 221.1 | 177.4 | 92.8 | 13 |
| FBDR+ CF+ sq-LNS | NP[2] | NP | NP | NP | NP | NP | NP | NP | NP | NP | NP | NP | NP | 0 |

**Supplemental Table 13. The optimal[3], best-case scenario[4] and worst-case scenario[5] of the baseline diet; the worst-case scenario for the food based diet recommendations and alternative combinations expressed as a percentage of the recommended nutrient intake in 6 to 8 mo. children of SNNP region**

| | Protein | Fat | Calcium | Vit. C | Vit B-1 | Vit B-2 | Niacin | Vit. B-6 | Folate | Vit. B-12 | Vit. A | Iron[6] | Zinc[7] | N[8] |
|---|---|---|---|---|---|---|---|---|---|---|---|---|---|---|
| Baseline[9] | 161.5 | 155.5 | 100 | 110.4 | 76.8 | 152.6 | 44.2 | 66.4 | 84.3 | 121.8 | 101.2 | 42.5 | 30 | 9 |
| best-case scenario[10] | 220.4 | 201.2 | 130.6 | 121.1 | 85.1 | 228.6 | 53.2 | 76.8 | 90.2 | 189.9 | 118.2 | 43.7 | 32.6 | 10 |

[1] Best alternative option

[2] NP= Combination not possible due to energy constraints

[3] The optimal diet formulated by using goal programming (Optifood module II).

[4] Each diet sequentially maximizes each micronutrient (Optifood module III)

[5] Each diet sequentially minimizes each micronutrient (Optifood module III)

[6] Recommended nutrient intake for iron assuming 5% absorption

[7] Recommended nutrient intake for zinc assuming low absorption

[8] Number of nutrient that cover at least 70% of the recommended nutrient intake in the worst-case scenario.

[9] Nutritionally best possible diet (Optifood module II)

[10] Diet sequentially maximizes each nutrient (Optifood module III).

| | | | | | | | | | | | | | | |
|---|---|---|---|---|---|---|---|---|---|---|---|---|---|---|
| worst-case scenario | 93.9 | 144.9 | 53.6 | 109.9 | 43.7 | 64.5 | 34 | 38.1 | 47.1 | 47.6 | 100.5 | 6.8 | 16.3 | 4 |
| FBDR[1] | 150.8 | 154.6 | 95.1 | 110.1 | 71.9 | 146.1 | 41.5 | 59.4 | 76 | 118.3 | 101.1 | 35.2 | 28.3 | 9 |
| CF3.5 s/wk.[2] | 100.9 | 145.3 | 54.3 | 112 | 47.9 | 66.2 | 37.4 | 40 | 67.4 | 47.6 | 100.6 | 6.5 | 16.9 | 4 |
| CF 7 s/wk. | 124.6 | 147.4 | 55.5 | 114.1 | 63.9 | 71.8 | 45.2 | 55 | 93.1 | 47.6 | 100.7 | 11 | 20.4 | 7 |
| MNP 3.5 s/wk. | 93.9 | 144.9 | 53.6 | 159.9 | 127.1 | 127 | 109 | 121.4 | 140.9 | 111.9 | 100.5 | 22.9 | 40.7 | 10 |
| MNP 7 s/wk. | 93.9 | 144.9 | 53.6 | 209.9 | 210.4 | 189.5 | 184 | 204.8 | 234.6 | 176.2 | 100.5 | 39 | 65.1 | 10 |
| sq-LNS 3.5 s/wk. | 93.7 | 166.5 | 88 | 159.8 | 83.1 | 110.7 | 80.2 | 76.9 | 92.1 | 83.1 | 150.5 | 18.6 | 61.2 | 11 |
| sq-LNS 7 s/wk. | 103.9 | 188.8 | 122.7 | 209.8 | 130.8 | 159.4 | 129.2 | 124.2 | 141.2 | 118.6 | 200.5 | 33.3 | 108.6 | 12 |
| FBDR+ CF 1 s/wk. | 157.9 | 155.4 | 95.5 | 110.7 | 76.6 | 147.8 | 43.7 | 63.8 | 83.5 | 118.3 | 101.2 | 26.6 | 29.3 | 9 |
| FBDR+ MNP 3.5 s/wk. | 150.8 | 154.6 | 95.1 | 160.1 | 155.2 | 208.6 | 116.5 | 142.7 | 169.8 | 182.5 | 101.1 | 51.2 | 52.7 | 11 |
| FBDR+ MNP 7 s/wk. | 150.8 | 154.6 | 95.1 | 210.1 | 238.5 | 271.1 | 191.5 | 226 | 263.5 | 246.8 | 101.1 | 67.4 | 77.1 | 12 |
| FBDR+ sq-LNS 1 s/wk. | 154.9 | 161.3 | 105.1 | 124.4 | 86.1 | 160.3 | 55.7 | 73.6 | 90.3 | 128.4 | 115.4 | 39.8 | 41.9 | 10 |
| FBDR+CF 1 s/wk. +MNP 3.5 s/wk. | 150.8 | 154.6 | 95.1 | 160.1 | 155.2 | 208.6 | 116.5 | 142.7 | 169.8 | 182.5 | 101.1 | 51.3 | 52.7 | 11 |
| FDBR+CF1 s/wk. +MNP 7 s/wk.[3] | 150.8 | 154.6 | 95.1 | 210.1 | 238.5 | 271.1 | 191.5 | 226 | 263.5 | 246.8 | 101.1 | 67.4 | 77.1 | 12 |
| FBDR+ CF s/wk. + sq-LNS s/wk. | NP[4] | NP | NP | NP | NP | NP | NP | NP | NP | NP | NP | NP | NP | 0 |

**Supplemental Table 14. The optimal[5], best-case scenario[6] and worst-case scenario[7] of the baseline diet; the worst-case scenario for the food based recommendations and alternative combinations expressed as a percentage of the recommended nutrient in 9 to 11 mo. children of SNNP.**

| | Protein | Fat | Calcium | Vit. C | Vit B-1 | Vit B-2 | Niacin | Vit. B-6 | Folate | Vit. B-12 | Vit. A | Iron[8] | Zinc[9] | N[10] |
|---|---|---|---|---|---|---|---|---|---|---|---|---|---|---|

[1] Set of food based dietary recommendations selected.

[2] s/wk=number of servings per week

[3] Best alternative option

[4] NP= Combination not possible due to energy constraints

[5] The optimal diet formulated by using goal programming (Optifood module II).

[6] Each diet maximizes each micronutrient (Optifood module III)

[7] Each diet minimizes each micronutrient (Optifood module III)

[8] Recommended nutrient intake for iron assuming 5% absorption

[9] Recommended nutrient intake for zinc assuming low absorption

[10] Number of nutrient to reach at least 70% of the recommended nutrient intake in the worst case scenario.

| | | | | | | | | | | | | | | |
|---|---|---|---|---|---|---|---|---|---|---|---|---|---|---|
| Baseline[1] | 162.1 | 138.2 | 100.6 | 136 | 101.5 | 137.6 | 66.2 | 132.9 | 115.6 | 100 | 170.9 | 76.6 | 36.1 | 11 |
| best-case scenario[2] | 211.8 | 192.8 | 130.6 | 163.5 | 121.7 | 219.5 | 86.5 | 191.4 | 161.4 | 174.7 | 185.8 | 81.3 | 40.4 | 12 |
| worst-case scenario | 84.1 | 121.3 | 50.9 | 102.5 | 48 | 63.9 | 32.6 | 52.9 | 49.6 | 44.8 | 93.8 | 10.6 | 15.5 | 4 |
| FBDR[3] | 121.2 | 127.6 | 97.4 | 124.5 | 70.4 | 128 | 49 | 116.3 | 93 | 81.1 | 149.9 | 49.8 | 24.3 | 10 |
| CF3.5 s/wk.[4] | 92.3 | 122.4 | 51.4 | 104.7 | 51.9 | 66.4 | 37 | 53.7 | 69.3 | 44.8 | 93.9 | 9.9 | 16.8 | 4 |
| CF 7 s/wk. | 108.2 | 124.4 | 52.1 | 106.8 | 63.3 | 69.2 | 43.3 | 55.3 | 91 | 44.8 | 94 | 11.3 | 19.5 | 5 |
| MNP 3.5 s/wk. | 84.1 | 121.3 | 50.9 | 152.5 | 131.3 | 126.4 | 107.6 | 136.2 | 143.3 | 109.1 | 93.8 | 26.7 | 39.9 | 10 |
| MNP 7 s/wk. | 84.1 | 121.3 | 50.9 | 202.5 | 214.6 | 188.9 | 182.6 | 219.6 | 237.1 | 173.4 | 93.8 | 42.9 | 64.3 | 10 |
| sq-LNS 3.5 s/wk. | 85.2 | 141.4 | 85.2 | 152.5 | 87.8 | 110.9 | 79.6 | 91.2 | 94.6 | 80.3 | 143.8 | 22.3 | 61.3 | 12 |
| sq-LNS 7 s/wk. | 90.6 | 161.9 | 119.5 | 202.5 | 131.8 | 158.1 | 127.4 | 130 | 140 | 115.8 | 193.8 | 34.2 | 107.9 | 12 |
| FBDR+ CF 3.5 s/wk. | 143.6 | 129.9 | 98.7 | 128.6 | 87.5 | 134.3 | 57.6 | 131.1 | 118.6 | 81.1 | 150 | 54.8 | 27.9 | 10 |
| FBDR+ MNP 3.5 s/wk. | 121.2 | 127.6 | 97.4 | 174.5 | 153.8 | 190.5 | 124 | 199.6 | 186.8 | 145.4 | 149.9 | 65.9 | 48.7 | 11 |
| FBDR+ MNP 7 s/wk.[5] | 121.2 | 127.6 | 97.4 | 224.5 | 237.1 | 253 | 199 | 283 | 280.5 | 209.6 | 149.9 | 82.1 | 73.1 | 13 |
| FBDR+ sq-LNS 1 s/wk. | 124.8 | 133.5 | 107.4 | 138.8 | 84.6 | 142.2 | 63.3 | 130.3 | 107 | 91.2 | 164.2 | 54.4 | 37.9 | 9 |
| FBDR+ sq-LNS 3.5 s/wk. | 134 | 148.5 | 132.4 | 174.5 | 120.2 | 177.7 | 99 | 165.7 | 142.7 | 116.6 | 199.9 | 65.9 | 71.9 | 12 |
| FBDR+CF3.5 s/wk. +MNP 3.5 s/wk. | 143.6 | 129.9 | 98.7 | 178.6 | 170.8 | 196.8 | 132.6 | 214.4 | 212.4 | 145.4 | 150 | 71 | 52.3 | 12 |
| FDBR+CF3.5 s/wk. +MNP 7 s/wk. | 143.6 | 129.9 | 98.7 | 228.6 | 254.2 | 259.3 | 207.6 | 297.8 | 306.1 | 209.7 | 150 | 87.1 | 76.6 | 13 |
| FBDR+ CF2 s/wk. + sq-LNS2 s/wk. | 141.6 | 140.9 | 118.1 | 157.1 | 109.3 | 160.5 | 83.1 | 153.1 | 136.1 | 101.4 | 178.5 | 61.9 | 53.7 | 11 |

[1] Nutritionally best possible diet (Optifood module II)

[2] Diet sequentially maximizes each nutrient (Optifood module III).

[3] Set of food based dietary recommendations selected.

[4] s/wk=number of servings per week

[5] Best alternative option

**Supplemental Table 15. The optimal[1], best-case scenario[2] and worst-case scenario[3] of the baseline diet; the worst-case scenario for the food based recommendations and alternative combinations expressed as a percentage of the recommended nutrient intake in 12 to 23 mo. children of SNNP region.**

| | Protein | Fat | Calcium | Vit. C | Vit B-1 | Vit B-2 | Niacin | Vit. B-6 | Folate | Vit. B-12 | Vit. A | Iron[4] | Zinc[5] | N[6] |
|---|---|---|---|---|---|---|---|---|---|---|---|---|---|---|
| Baseline[7] | 250.7 | 109.4 | 100 | 122.5 | 109.6 | 170.8 | 96 | 117.5 | 100 | 100 | 141.6 | 128.1 | 62.1 | 1 |
| best-case scenario[8] | 283.4 | 173 | 114.1 | 180.1 | 125.4 | 206.8 | 121.8 | 216.5 | 130.3 | 135.2 | 182.3 | 201.6 | 65.1 | 1 |
| worst-case scenario | 89.9 | 88.1 | 38.4 | 91.4 | 42.8 | 54.4 | 26.9 | 61 | 33.9 | 31.8 | 83.6 | 33 | 17 | 4 |
| FBDR[9] | 243.6 | 113.6 | 77.7 | 93.4 | 100 | 159.6 | 81.4 | 101.1 | 96.6 | 120.9 | 111.6 | 108.4 | 57.9 | 12 |
| CF3.5 s/wk.[10] | 102.6 | 88.9 | 38.7 | 94.6 | 45.6 | 56.7 | 29.5 | 52.5 | 49.6 | 31.8 | 83.7 | 33 | 19.4 | 4 |
| CF 7 s/wk. | 121.5 | 90.4 | 39.2 | 97.8 | 50.7 | 60 | 33.5 | 53.3 | 65.4 | 31.8 | 83.9 | 34.1 | 22.4 | 4 |
| MNP 3.5 s/wk. | 89.9 | 88.1 | 38.4 | 141.4 | 92.8 | 104.4 | 76.9 | 111 | 83.9 | 81.8 | 83.6 | 58.8 | 41.7 | 10 |
| MNP 7 s/wk. | 89.9 | 88.1 | 38.4 | 191.4 | 142.8 | 154.4 | 126.9 | 161 | 133.9 | 131.8 | 83.6 | 84.7 | 66.4 | 11 |
| sq-LNS 3.5 s/wk. | 90.9 | 103.6 | 65.5 | 141.4 | 66.4 | 91.3 | 57.2 | 70 | 57.9 | 59.4 | 133.6 | 51.9 | 63.6 | 6 |
| sq-LNS 7 s/wk. | 94.8 | 119.2 | 93 | 191.4 | 90.1 | 129 | 87.9 | 99 | 81.9 | 87 | 183.6 | 71.8 | 110.3 | 13 |
| FBDR+ CF 1 s/wk. | 252.5 | 114.4 | 78.2 | 94.4 | 104.2 | 161.7 | 83.7 | 105.1 | 102.5 | 120.9 | 111.6 | 111.9 | 59.5 | 12 |
| FBDR+ MNP 3.5 s/wk.[11] | 243.6 | 113.6 | 77.8 | 143.4 | 150 | 209.6 | 131.4 | 151.1 | 146.6 | 170.9 | 111.6 | 134.3 | 82.6 | 13 |
| FBDR+ MNP 7 s/wk. | 243.6 | 113.6 | 77.8 | 193.4 | 200 | 259.6 | 181.4 | 201.1 | 196.6 | 220.9 | 111.6 | 160.1 | 107.3 | 13 |
| FBDR+ sq-LNS 1 s/wk. | 254.6 | 120.8 | 92.9 | 110.3 | 111.1 | 178.1 | 93.3 | 112.7 | 106.2 | 134.5 | 127.1 | 125.5 | 73.7 | 13 |
| FBDR+CF 1 s/wk. +MNP 3.5 s/wk. | 252.2 | 114.4 | 78.2 | 144.4 | 154.2 | 211.7 | 133.7 | 155.1 | 152.5 | 170.9 | 111.6 | 137.8 | 84.2 | 13 |
| FDBR+CF1 s/wk. +MNP 7 s/wk. | 252.2 | 114.4 | 78.2 | 194.4 | 204.2 | 261.7 | 183.7 | 205.1 | 202.5 | 220.9 | 111.6 | 163.6 | 108.8 | 13 |
| FBDR+ CF1 s/wk. + sq-LNS1 s/wk. | 259.6 | 122 | 94.4 | 121 | 113.2 | 179.3 | 97 | 115.4 | 110.4 | 135.7 | 137.7 | 131.2 | 70.4 | 13 |

[1] The optimal diet formulated by using goal programming (Optifood module II).

[2] Each diet maximizes each micronutrient (Optifood module III)

[3] Each diet minimizes each micronutrient (Optifood module III)

[4] Recommended nutrient intake for iron assuming 5% absorption

[5] Recommended nutrient intake for zinc assuming low absorption

[6] Number of nutrients to cover at least 70% of the recommended nutrient intake in the worst-case scenario

[7] Nutritionally best possible diet (Optifood module II)

[8] Diet sequentially maximizes each nutrient (Optifood module III).

[9] Set of food based dietary recommendations selected.

[10] s/wk=number of servings per week

[11] Best alternative option

**Online Supporting Material**
